# Underlying biological mechanisms of emotion dysregulation in bipolar disorder

**DOI:** 10.3389/fpsyt.2025.1552992

**Published:** 2025-05-09

**Authors:** Buse Beril Durdurak, Isabel Morales-Muñoz, Angharad N. de Cates, Chantelle Wiseman, Matthew R. Broome, Steven Marwaha

**Affiliations:** ^1^ Institute for Mental Health, University of Birmingham, Edgbaston, Birmingham, United Kingdom; ^2^ Coventry and Warwickshire NHS Partnership Trust, Coventry, United Kingdom; ^3^ Department of Psychiatry, University of Oxford, Oxford, United Kingdom; ^4^ East Birmingham Early Intervention in Psychosis Service, Birmingham Women’s and Children’s NHS Foundation Trust, Birmingham, United Kingdom; ^5^ Specialist Mood Disorders Clinic, The Barberry Centre for Mental Health, Birmingham and Solihull NHS Trust, Birmingham, United Kingdom

**Keywords:** bipolar disorder, emotion regulation, biological, mechanisms, underlying

## Abstract

Difficulties with emotion regulation (ER) are a key feature of bipolar disorder (BD) contributing to poor psychosocial and functional outcomes. Abnormalities within emotion processing and regulation thus provide key targets for treatment strategies and have implications for treatment response. Although biological mechanisms and ER are typically studied independently, emergent findings in BD research suggest that there are important ties between biological mechanisms and the disturbances in ER observed in BD. Therefore, in this narrative review, we provide an overview of the literature on biological mechanisms underlying emotional dysregulation in BD including genetic and epigenetic mechanisms, neuroimaging findings, inflammation, hypothalamic-pituitary-adrenal (HPA) axis dysfunction, neuroplasticity and brain-derived neurotrophic factor (BDNF), and circadian rhythm disturbances. Finally, we discuss the clinical relevance of the findings and provide future directions for research. The continued exploration of underlying biological mechanisms in ED in BD may not only elucidate fundamental neurobiological mechanisms but also foster advancements in current treatment strategies and the development of novel targeted treatments.

## Introduction

Bipolar Disorder (BD) is a common and highly debilitating condition with recurrent mood episodes and subsyndromal mood symptoms (1, 2). Most contemporary treatment guidelines are designed around an episodic model of BD, emphasizing the management and prevention of acute episodes (3). However, mood in people with BD is often labile (1) and can fluctuate considerably even when euthymic (4), in contrast to the classical episodic presentation (5).

Emotion Dysregulation (ED) is a multidimensional transdiagnostic construct encompassing a wide range of mental disorders (6). In the literature, the term has been used interchangeably with mood instability, affective lability, affective dysregulation, and mood swings (7–9). Although no agreed-upon definition of the conceptual core of ED exists (10), one definition is that ED is defined as patterns of emotional experience and expression that interfere with goal-directed activity (11). Emotion regulation (ER) strategies are generally classified as adaptive or maladaptive based on their effectiveness and their positive or negative effects on mood (12). Difficulties in managing negative emotions, along with the use of maladaptive coping mechanisms like catastrophizing, self-blame, and rumination, are linked to higher rates of mental health issues (12), including BD (13).

### Emotion dysregulation in bipolar disorder

People living with BD may have difficulties in ER across various dimensions regardless of current mood episodes (14). ER difficulties in patients with BD may then result in emotional extremes and mood instability, eventually contributing to the episodic mood states of the condition (15–18). ED contributes considerably to functional impairments and relapse risk (19, 20). For example, ED may increase the severity of manic symptoms and residual depressive symptoms (21). Additionally, people with BD may have difficulties in facial emotion recognition (22), feel emotions more intensely (23) and have stronger elevations of negative emotion as compared to positive affectivity problems (17). ED in BD may also show poor acceptance and differentiation of emotion, as well as difficulties in inhibiting impulsive actions, using appropriate regulation strategies to modulate emotions, and engaging in adaptive behaviours to achieve a desired goal when experiencing negative mood (14). Patients with BD also tend to feel less confident in their ability to use adaptive emotion regulation strategies (24). As such, findings have indicated that in the face of a given emotion state, those with BD report more difficulty controlling their speech and behaviour (23, 25). This phenomenon is known as emotion-related impulsivity (25), and individuals with BD report significantly greater concern about this specific type of impulsivity compared to other forms, even during periods of remission (23). Higher levels of emotion-related impulsivity have been linked to increased suicidality and aggression in individuals with remitted BD I (25). Unsurprisingly, this form of impulsivity is also strongly associated with significantly lower quality of life and poorer functional outcomes in individuals with BD I (23, 26, 27).

Patients with BD may also show decreased emotional reactivity in positive social scenarios, impaired ability to down-regulate positive emotion, as well as a specific deficit in the ability to recognise surprised facial displays of emotion (28). Poor access to ER strategies can predict depressive propensity in BD, while difficulty in controlling impulses may predict trait (hypo)mania (14). More specifically, increased sensitivity to reward and deficient emotion processing and regulation in BD may amplify affect, thereby triggering a spiral into both manic and/or depressive states (29). Many of the facets of emotionality can be observed before onset among those at risk for the disorder (30). Further, recent findings suggest that abnormal ER may serve as an endophenotype for BD (31). Specifically, difficulties with ER are observed not only during acute episodes (32, 33) but also during periods of remission (34), as well as in genetically predisposed, unaffected relatives of individuals with BD (35). Indeed, previous functional magnetic resonance imaging (fMRI) studies have shown abnormal fronto-limbic responses during emotional reactivity and regulation in both BD patients and high-risk groups (36–38). Further, a systematic review found that not only patients with BD but also their unaffected relatives exhibit behavioural and neural difficulties with emotion processing and regulation. This includes impairments in the recognition of facial displays of emotion, reduced ability to successfully down-regulate positive emotions, and increased cognitive interference of emotional stimuli (29).

### Emotion dysregulation in bipolar disorder compared to other mental illnesses

When compared to other psychiatric diagnoses, a meta-analysis found that people with BD were quite similar to patients with Major Depressive Disorder (MDD) in both overall ED and adopted ER strategies, although they showed more positive rumination and risk-taking behaviours (13). Patients with BD exhibited a lower degree of ED when compared with Borderline Personality Disorder (BPD) patients, as they adopted more adaptive ER strategies and fewer maladaptive ones (13). A systematic review found that compared to both clinical and non-clinical controls, people with BD may also endorse putatively maladaptive strategies for regulating negative affect such as rumination, self-blame, suppression and catastrophising more strongly than non-clinical controls, but have a similar ER profile to people with unipolar depression (39). The review also found that although dampening positive affect was higher in BD than controls, it did not distinguish BD and MDD (39).

### Potential utility of understanding pathophysiological underpinnings of emotional dysregulation in bipolar disorder

Psychiatric diagnoses and symptomatology alone cannot fully capture the complexity of ED in BD, emphasizing the need for broader considerations (40). Contemporary classifications of mental disorders remain primarily based on clinical features (41), leading many individuals with mental illness to transition through multiple diagnostic categories over their lifetime (42). The absence of objective markers to confirm diagnoses often delays accurate treatment and early interventions, as overlapping symptoms (e.g., anxiety, insomnia, low mood, suicidal ideation, impulsivity) are common across various disorders (43). Moreover, these classifications fail to uncover the fundamental mechanisms driving the syndromes (44), underscoring the urgent need for objective biomarkers to refine diagnostic evaluations and predict illness trajectories (45).

BD is a highly heritable condition with a complex aetiology (46, 47). Its onset is thought to result from the interplay between genetic susceptibility and environmental factors that predispose, precipitate, and perpetuate the disorder (46). Emerging research highlights the role of genetics, environmental interactions, and prodromal features preceding episodes of (hypo)mania or depression in BD (48). Evidence suggests that BD involves alterations in numerous biological systems, including disrupted structural and functional brain connectivity (49–51), oxidative stress (52), mitochondrial dysfunction (53), inflammation (54), circadian rhythm disturbances (55), dopamine dysregulation (56), neurotrophic system abnormalities (e.g., elevated brain-derived neurotrophic factor levels), and calcium signaling disruptions (57–59).

Many of these biological mechanisms are also likely contributors to ED in BD. Brain regions critical to ER, such as the prefrontal cortex and limbic system, exhibit altered activation patterns in BD (38). fMRI studies have reported abnormal fronto-limbic responses during emotional reactivity and regulation in individuals with BD and those at high risk (37). Consequently, deficits in affective cognition (i.e., hot cognition) are increasingly recognized as core components of BD’s neurocognitive profile and potential treatment targets (60, 61). Affective cognition encompasses emotionally charged processes such as emotional processing and regulation, perceptual and attentional biases, feedback sensitivity, emotional decision-making, and reward-punishment processing (60, 62). Further, beyond cognition, multiple biological factors influence emotional processes in BD, including genetics (63), epigenetics (64), inflammation (65), hypothalamic-pituitary-adrenal axis dysfunction (66), brain-derived neurotrophic factor abnormalities (67), and circadian disruptions (68, 69).

To advance our understanding of ED in BD, precise definitions and a deeper exploration of its neural, molecular, and genetic mechanisms are essential (4). However, the relationship between BD and ED remains poorly understood. Given the pivotal role of ED in BD, it is imperative to elucidate its biological underpinnings. This narrative review aims to evaluate and synthesise current knowledge on the biological mechanisms involved in ER processes in BD, providing insights into the biological basis of ER in this disorder and identifying avenues for future research and intervention development.

## Genetic and epigenetic mechanisms

### Genetics

BD involves numerous risk loci, with most lead single nucleotide polymorphisms (SNPs) being noncoding and having small effect sizes (70). Findings so far highlight at least three genes; synaptic plasticity proteins (e.g., ANK3, located on chromosome 10q21.2; 71–73), ion channels (e.g., CACNA1C, located on chromosome 12p13; 74, 75), and TRANK1, a protein of unknown function that resides on chromosome 3p22 (76, 77).

Regarding ED, the genes most associated with this condition have previously been found to be associated with psychiatric disorders including BD (63). For example, a study examining genetic risk associated with ED found an overrepresentation of genes from the calcium signalling pathway and inflammation (i.e., IL2RA), also associated with risk for many psychiatric disorders including BD (63). Likewise, another study investigating genetic factors underlying emotional reactivity in BD patients found an association between ER and genetic variants in BD (78). The strongest associated SNP located in the 11q14 region has been implicated in nervous system development and the functional pathway of calcium ion binding (78). The second SNP retained, rs2064689 in the 1p31 region was located in the intron region of the interleukin 23 receptor precursor gene (i.e., IL23R NM_144701.2), which has also been implicated in inflammatory processes (78).

### Epigenetic mechanisms

Histone deacetylase (HDAC) inhibitors have been shown to control epigenetic programming associated with the regulation of cognition and behaviour (79, 80). Genetic association and clinical pharmacology have been used to identify a potential role for HDACs in BD (79). Inhibition of class I or II HDACs can protect neurons from mitochondrial oxidative stress induced damage frequently observed in BD (79, 81, 82). A systematic analysis of GWAS studies found that HDAC2 may be linked to increased genetic risk for BD through its involvement in the development of the amygdala, nucleus accumbens and hippocampus and alterations in these regions could increase BD risk (83). Additionally, pharmacological treatments for BD act on HDACs (64, 84). Specifically, valproic acid has the ability to promote gene expression through inhibition of class I and II HDACs activity and increase of acetylated histone H3 and acetylated histone H4 levels (85–87), lithium downregulates HDAC1 at the translational level by targeting HDAC1 messenger RNA (88), and lamotrigine increases histone acetylation levels *in vitro* (89). Regarding ED, a study using *in vivo* imaging of HDAC- specific radiotracers in individuals with BD found that altered levels and activity of HDACs were related to attention and ER, further suggesting a role for HDACs in BD pathophysiology (64).

## Structural and functional brain alterations

ER involves complex neural pathways integrating cognitive control and emotional responses (25). These pathways are categorized into implicit (automatic) and explicit (effortful) regulation (90), both of which involve distinct but interconnected brain regions. Individuals with BD exhibit abnormalities in these circuits, contributing to ED (91).

Neuroimaging studies have identified structural and functional abnormalities in BD, particularly within the limbic system (amygdala, hippocampus, and basal ganglia) and the prefrontal cortex (PFC). Meta-analyses of fMRI studies highlight hyperactivity in limbic structures, including the amygdala and hippocampus, alongside hypoactivity in the right inferior frontal gyrus (IFG), a key region involved in cognitive control over emotions (37). Similarly, another meta-analysis examining abnormal brain activation during ER in BD found clusters of hyperactivation in subcortical structures and limbic regions, including the caudate, amygdala, and parahippocampal gyrus, as well as cortical frontal structures such as the precentral and frontal gyri (92). Conversely, hypoactivation was observed in cortical regions such as the anterior cingulate and inferior frontal gyri in response to facial stimuli.

White matter (WM) abnormalities also contribute to ER deficits in BD (93). Studies have reported reduced fractional anisotropy in WM tracts connecting the PFC, dorsolateral PFC (dlPFC), ventrolateral PFC (vlPFC), medial PFC (mPFC), and left parietal cortex. Increased mean and radial diffusivity in the superficial WM of the right frontal cortex further supports structural connectivity disruptions in BD. These findings align with reports of microstructural abnormalities in the corpus callosum and corticospinal tract, both of which are associated with impaired emotional and cognitive functioning (94).

Youth at risk for BD also exhibit altered neural activation patterns during emotional processing (95). For example, increased right rostral anterior cingulate cortex (ACC) activity in response to happy stimuli and heightened amygdala-to-left caudal ACC connectivity in response to fearful stimuli have been observed (95). These findings suggest that neural circuits underlying ER are already compromised before illness onset, reinforcing the role of brain-based markers in identifying at-risk populations.

Overall, research underscores that BD involves widespread disruptions in ER-related neural circuits (25). FMRI studies introduced a new perspective to studying the neural foundations of psychological disorders associated with ER, offering insights into potential variations in brain network dynamics related to mood or cognition, as well as in their regulation by executive control or reward-related functions (4, 96). However, much of the existing literature is correlational, limiting causal inferences. Future studies should focus on elucidating the interplay between brain alterations, ER, and functional outcomes using longitudinal designs (25). Understanding these mechanisms will inform targeted interventions aimed at improving ER in BD (25).

### Disruptions in neural circuits involved in ER in BD and other mental health illnesses

Since ED is a transdiagnostic construct, disruptions in neural circuits involved in emotional processing are found across a wide range of conditions (97). For example, a meta-analysis with a transdiagnostic sample identified hyperactivity in the amygdala, hippocampal/parahippocampal gyri, and dorso-medial thalamus, alongside reduced prefrontal cortex activity, not only in BD but also in anxiety, depressive, and substance use disorders, (98). Likewise, another study found common neural variability correlates of ED in the fronto-limbic system in BD, BPD and ADHD (97). Dysfunctions in the prefrontal cortex region has been linked to perseverative negative bias in depression (99), and this region may also contribute to rumination observed across disorders (98, 100).

The ventromedial PFC is a key neural region in social and affective function (101). It is also considered central to the pathophysiology of mood and anxiety disorders (102). For example, a meta-analysis by Hiser and Koenigs (103) examined the associations between different subregions of the ventromedial prefrontal cortex (vmPFC) and psychiatric disorders. Their findings indicated that dysfunctions in distinct vmPFC subregions are linked to various mental illnesses, including BD, major depressive disorder, and post-traumatic stress disorder. Specifically, the study found that addiction, BD, and ADHD-related neural activity overlap with the decision-making subregion of the vmPFC, while PTSD, schizophrenia, and social anxiety disorder exhibit dysfunctions in both the decision-making and social processing subregions. These findings emphasize the role of vmPFC dysfunction in emotional and cognitive dysregulation across psychiatric conditions (98, 103–105).

On the other hand, notable differences between disorders may reflect underlying psychopathological distinctions. For example, the previously mentioned meta-analysis above (98) found that while hyperactivity in the amygdala, hippocampal/parahippocampal gyri, and dorso-medial thalamus, alongside reduced prefrontal cortex activity was observed in BD, anxiety, depressive, and substance use disorders, there were also differences, suggesting that therapeutics tuned to specific disruptions within the network may be preferentially effective for some neurobehavioral phenotypes. They found that while anxiety disorders were marked by pronounced amygdala/hippocampal hyperactivation, whereas BD was marked by ventrolateral prefrontal cortex hypoactivation, unipolar disorders by amygdala hyperactivation, and substance use disorders by ventrolateral prefrontal cortex hypoactivation.

The challenge of differentiating between BD and MDD in clinical settings has prompted researchers to identify differences in the pathophysiological mechanisms underlying BD and MDD (106). While both patients with MDD and BD exhibit predominantly decreased fronto-limbic connectivity (107), their disorder-specific pathophysiological mechanisms may differ (33). For example, different activation patterns in neural networks including the amygdala, ACC, PFC, and striatum during emotion-, reward-, or cognition-related tasks have been reported between BD and MDD (108). More specifically, a stronger functional connectivity pattern in BD was pronounced in default mode and in frontoparietal networks and brain regions including the PFC, ACC, parietal and temporal regions, and thalamus compared to MDD. Lastly, BD showed reduced integrity in the anterior part of the corpus callosum and posterior cingulum compared to MDD. Another fMRI study investigating ER differences in medication-free patients with MDD and BD revealed that these differences are mood-state dependent (109). During remission, patients with BD, but not those with MDD, exhibited impaired ER across emotions, which was associated with increased dorsolateral PFC activity. However, during depressive episodes, MDD and BD patients differed in their ER of happy versus sad emotions. BD patients in a depressive phase demonstrated normal ER for happy stimuli but impairments for sad stimuli, whereas MDD patients rated sad pictures as less negative and happy pictures as less positive than BD patients. These ER differences were linked to activity in the dorsolateral PFC and rostral ACC.

Similar to the overlap between BD and MDD, BD and BPD share neuroimaging findings in addition to behavioural similarities (110). Shared features include aberrant connections between limbic regions and the PFC (111–113), reduced brain glucose metabolism in the brainstem, insula, and frontal white matter (114), gray matter alterations (115), and dysregulation of the HPA axis (116–119). Despite these similarities, key differences exist. For instance, a recent study found that patients with BPD had significantly increased WM components mostly regions in the left parietal and frontal lobes (120). This white matter component largely overlaps with the network involved in top-down cognitive control of emotions (121–124). The authors suggest that the increased white matter component in BPD may reflect attempts by these patients to exert control over their emotional reactions (120).

Lastly, BD and ADHD are neuropsychiatric disorders with a broad overlap in psychopathology, and possibly, pathophysiology (125). Both disorders are highly comorbid (126), and share several common symptoms, including impulsivity, psychomotor agitation, poor sleep, and cognitive impairments, including inattention and distractibility (125). The cognitive deficits observed in BD and ADHD follow a similar pattern across multiple cognitive domains (125), though their severity and presentation may vary between individuals (127, 128). Regarding ED, brain regions implicated in ED in BD are also implicated in ADHD (46, 129) including reduced gray matter volumes in the right insula and ACC (130). However, some neurobiological differences have been reported. For instance, ADHD patients exhibit reduced activation in the ventrolateral prefrontal cortex (VLPFC) compared to healthy controls (HC) and paediatric BD patients (131). Similarly, youth with bipolar spectrum disorders display abnormally heightened functional connectivity between the amygdala and regions of the ventral prefrontal cortex during emotion processing, a pattern not observed in youth with ADHD or HC (132). Furthermore, adolescents with BD show increased connectivity between the affective and ventral attention networks, as well as within the attention network, compared to those with ADHD (133).

Future studies should investigate whether altered functional connectivity serves as an endophenotype of BD and whether it can help identify youth with externalizing symptoms who may later develop bipolar spectrum disorders (132).

Overall, these differences suggest that while pleiotropy and inadequate validity of diagnostic categorization remain challenges in psychiatry, the multifactorial nature of causation leaves room for unique combinations of causal factors contributing to each disorder (42).

### Investigating the effects of lithium on disturbances in limbic and emotion regulation circuits

Lithium has long been considered a key and gold-standard pharmacological treatment for BD (134, 135). However, the specific processes through which its therapeutic effects such as mood stabilisation are affected and maintained remain unclear (136, 137). Structural neuroimaging studies in patients with BD have suggested that treatment with lithium normalizes or increases gray matter volume in the anterior cingulate, subgenual cingulate cortex, amygdala and hippocampus, which are brain regions involved in ER (138, 139). Recent research investigating lithium’s effect on ER in BD (15) found that lithium’s effect on active reappraisal and emotional processing of negative images was seen in areas of the fronto-parietal and limbic network, and in superior and medial temporal structures. Further, they found that within the fronto-parietal network, lithium decreased activation in prefrontal areas (left anterior PFC or rostra-lateral PFC, and right superior frontal gyrus) and posterior parietal areas (left angular gyrus), during reappraisal. There were also connectivity changes within prefrontal and limbic areas following lithium administration. Further, response to lithium treatment in paediatric BD was associated with normalization of white matter microstructure in regions associated with emotion processing (140). Likewise, a study examining how treatment with quetiapine or lithium impacts limbic and ER brain circuitry in youth with BD found both treatments had a normalizing effect on brain function, although quetiapine induced a more rapid and widespread normalization of brain function (141). Lastly, a review examining fMRI studies evaluating the impact of lithium on brain functional activity and connectivity in patients with BD found that selective abnormalities in neural circuitries supporting emotional processing and regulation improve during lithium treatment in BD (142). However, further research is needed to understand the connection between ER circuitry and the therapeutic action of lithium in BD. Investigating the regional neurophysiological effects of antimanic treatments can offer insights into the mechanisms underlying their clinical efficacy at the systems level (141). This understanding may also inform strategies for testing new compounds and, in the long term, support clinical decision-making regarding the initiation or adjustment of therapeutic interventions (141).

## Inflammation

Inflammation is a complex biological process involving cytokine cascades, immune cell responses, and elevated levels of acute-phase proteins and complement factors (59). Increasing evidence indicates that chronic, low-level inflammation in the body and brain (i.e., neuroinflammation) contributes to the pathophysiology of BD (143, 144), both as a state and a trait feature (48, 145) and can worsen the disease progression (146). The connection between BD and inflammation is further supported by shared genetic polymorphisms and gene expression patterns (147).

Chronic inflammation also impacts brain functions, such as the monoaminergic system, which plays a role in ER (78). A systematic review found that difficulty regulating emotion was associated with elevated inflammatory markers, while effective ER was linked to lower inflammation levels (148). Specifically, use of cognitive reappraisal has been associated with lower levels of C-reactive protein (CRP; 149) and interleukin-6 (IL-6; 150) whereas increased inflammation, specifically CRP, tumor necrosis factor-α (TNF-α), and interferon-γ (IFN-γ), have been linked with routine expressive suppression use (149, 151–153). Negative self-referential processes (e.g., self-criticism, negative thoughts, and rumination) have also been linked to altered peripheral inflammatory markers and cortisol responses in healthy adults (148, 154–156).

Regarding ED in BD, research suggests that inflammation can affect brain structures, which might lead to a dysregulated stress response and, possibly, to ED disorders such as BD (110, 157). While the causal relationship between inflammation and brain imaging abnormalities in BD remains uncertain (65), inflammation’s neurotoxic effects may impair neural circuits involved in ER, reward processing, motivation, emotional control, and cognition (158–161). For example, higher inflammatory biomarkers have been identified in BD patients’ left hippocampus (162) and higher soluble IL-6 receptor levels were associated with increased functional connectivity between the medial PFC and several subcortical limbic structures, including the amygdala, pallidum, hippocampus, and insula (163). Moreover, high-sensitivity CRP has been proposed as a potential objective marker of ER difficulties in BD (164). In one study, remitted BD patients with emotional hyper-reactivity exhibited higher levels of low-grade inflammation (e.g., high-sensitivity CRP), hypertension, impaired glucose metabolism, and a greater number of suicide attempts compared to those without hyper-reactivity (165). Elevated high-sensitivity CRP levels were also observed in BD patients with emotional hypo-reactivity (164).

Evidence from neuroimaging studies further supports the role of fronto-limbic network disruptions in ER difficulties associated with BD (166), including abnormalities in WM microstructure (15). Studies also show an association between neuroinflammation (e.g., circulating pro-inflammatory cytokines) and WM integrity (167). The balance of immune cell subsets, such as T cells and cytokine-producing natural killer cells, may be crucial for maintaining the brain’s structural and functional integrity in BD (168), while also contributing to the chronic low-grade inflammation observed in the disorder (169). For example, circulating natural killer cell counts, and particularly cytokine-producing natural killer cells, positively associated with Diffusion Tensor Imaging (DTI) measures of WM integrity and corticolimbic functional response and connectivity during emotional processing (170). More specifically, the study found an effect of CD56+TNFα+, CD56+INFγ+, and CD56+GMCSF+ on functional connectivity and response in brain regions which exert a crucial role in defining emotional experience and mood control such as dorsolateral pre-frontal cortex, hippocampus, precuneus, cuneus and amygdala connections with parahippocampal gyrus and temporal pole (93, 170). Lastly, they found a positive effect of lithium on cytokine-producing natural killer cell counts (170), which may in return reduce ED (15).

Although the connection between inflammation and ED in BD requires further exploration, preliminary evidence suggests a relationship between specific inflammatory markers and both behavioural changes and neuroanatomical alterations in regions involved in ER (157). Utilising biomarkers during the early stages of BD may aid in diagnostics and provide potential therapeutic avenues (171). Notably, anti-inflammatory treatments have shown promise in alleviating BD symptoms, particularly depressive symptoms (172–175). Additionally, antipsychotics and lithium have been observed to reduce the expression of several inflammatory genes, which are more frequently upregulated in monocytes from BD patients and their offspring than in healthy individuals (176, 177). Further research exploring the interplay between inflammation and ER in BD, particularly through longitudinal studies incorporating multiple timepoints, is warranted (110). Evaluating dimensions related to ER (e.g., levels of emotional reactivity) alongside accessible biomarkers such as blood pressure, CRP, and glycaemia, in addition to monitoring mood symptoms in BD patients, may help implement interventions more aligned with the underlying pathophysiology of BD (178). Finally, psychosocial interventions targeting inflammation through ER skills, such as cognitive-behavioral therapy and mindfullness, may serve as effective strategies for symptom management (148, 179).

## Hypothalamic-pituitary-adrenal axis dysfunction

Hypothalamic–pituitary– adrenal axis (HPA) is one of the main biological systems involved in response to stress and shaping the central nervous system (CNS) structures along the lifespan (180). Chronic stress and HPA axis dysregulation can disrupt inhibitory control, leading to heightened emotional responses (181). HPA axis dysregulations are also considered to play a major role in the pathophysiology and progression of BD (182). For example, state and trait hyperactivity of the HPA axis is associated with BD and abnormalities of glucocorticoid signalling are found in several key brain areas which in return may contribute to ED observed in BD (180). Disturbance in the HPA axis could also increase treatment resistance and relapse, worsen illness outcome, cause cognitive deficits and exacerbate depressive symptoms (183).

The link between ER and HPA-axis function has not been extensively investigated. Some research, however, indicates that positive ER strategies, such as problem-solving, are associated with lower cortisol levels over time (184) and with increased immediate cortisol responses (185, 186). It is still uncertain whether HPA-axis dysfunctions in BD are directly related to issues with ER (66). However, trauma experienced in childhood or later in life may influence both HPA-axis function and ER abilities (66). Both childhood trauma and chronic stress have been associated with dysregulations of the HPA axis (187) and alterations across various stages of ER (66), including emotion-related attentional biases and reward processing disturbances (188, 189).

Childhood trauma also plays a pervasive role in the development and course of BD (190, 191). For example, individuals with BD report higher rates of ED and individuals with BD who report childhood trauma also endorse higher rates of ED and impulsivity compared with those without trauma histories (191–193) and show poor response to lithium (192, 193). Less genetic risk may be needed to develop a more unstable form of BD when exposed to childhood maltreatment (194). This may suggest that some ER patterns seen in individuals with BD may not be unique to BD but could instead reflect the effects of early trauma (66) as childhood adversity, particularly emotional abuse, is associated with affective lability and ER in adulthood across psychiatric disorders (195). Research further suggests that childhood trauma, particularly emotional abuse, is associated with increased affective instability and less adaptive ER in BD (196–198). This could explain the relationship between childhood trauma and poor BD outcomes (191). Thus, it is important to consider contextual factors such as trauma that might contribute to the overlap between biological mechanisms (e.g., HPA-axis dysfunctions) and ER difficulties in BD before concluding that biological mechanisms are truly a central driver for other outcomes (25). Future research on BD-specific ER should account for the influence of early trauma, which may have either an additive effect or an interactive one, where childhood trauma combined with a genetic predisposition to BD shapes distinct ER patterns (66).

## Neuroplasticity and brain-derived neurotrophic factor

Brain-derived neurotrophic factor (BDNF) is a multifunctional neurotrophin with numerous functions in the brain such as neuronal plasticity and maintenance of the CNS (199), modulating reactivity to stress (200, 201). BDNF has been implicated in the pathophysiology of several psychiatric disorders (202), including BD (203, 204) even in at-risk stages (205). Research shows that the pattern of neurotrophin expression during mood episodes changes (206–208). For example, serum BDNF levels decrease during manic and depressive episodes in both treated and drug-free subjects when compared to normal controls and to unipolar depression (209). In relation to ER in BD, BDNF could serve as an objective marker for assessing ER interventions in BD (210).

One of the functional SNP of the BDNF gene in humans is in exon 11 and results in an amino-acid substitution from valine (Val) to methionine (Met) at codon 66 (rs6265, also known as Val66Met). Associations have been reported between ED and the 66Met allele of the BDNF, which is associated with reduced amygdala habituation to emotional stimuli during fMRI (211). Abnormal neural responses of amygdala networks during habituation to repeated emotional stimuli may be a putative endophenotype of psychiatric disorders characterized by ED (211). The Met allele may also interact with childhood abuse to predict an impaired ability to downregulate negative affect (212). Patients with BD who carry the Met allele at the BDNF Val66Met polymorphism have been shown to be more likely to develop depressive episodes following stressful life events than Val allele homozygous (67).

## Chrono-rhythmic disturbances

Circadian rhythm disturbances are integral to the pathophysiology of BD (213). For example, many circadian genes have previously been associated with BD, including ARNTL1, CLOCK, NPAS2, NR1D1, PER3, RORB, TIMELESS (214–218). In addition, some evidence suggests that genes involved in circadian rhythm regulation (e.g., CRY1 variants) may be associated with increased risk of relapse of depressive episodes in patients with BD (219). Disruptions in sleep-wake cycles, social rhythms, and hormonal fluctuations can also significantly influence ER (220).

### The role of the suprachiasmatic nucleus

Many individuals with BD have circadian rhythm alterations of sleep-wake, activity, melatonin, and other hormones even in euthymic periods (221). Circadian rhythms (e.g., sleep/wake cycles, body temperature control, and the release of neurotransmitters and hormones) are produced and coordinated by a central biological clock, the SCN, which is located in the anterior hypothalamus (222). Dysregulation in these rhythms can stem from enduring disruptions within the SCN’s own pacemaking functions or from impairments in how the SCN aligns with external cues (223). Although weakly, multiple SNPs in the genes encoding the core components of the molecular clock have been demonstrated to be associated with schizophrenia, BD and MDD, suggesting a causal role for clock dysfunction in psychiatric disorders (224). Furthermore, when individuals with or at-risk for BD experience life events that alter their daily routines or social rhythms (e.g., changes in sleep, meals, or work schedules), these disturbances can unsettle their circadian patterns, triggering physical symptoms that may eventually escalate into mood episodes (68).

### Sleep-wake cycles

Delayed sleep-wake and activity patterns are commonly seen in the depressive phase of BD (55, 225–229), particularly in adolescents and young adults (226, 227, 229–231). Changes in sleep phase, structure and duration are also reported across all mood states in BD (232). Several studies using an interview-based assessment of biological rhythm disturbances including sleep and activity have reported that young adults with BD have greater biological rhythm disruption than healthy controls (233, 234).

Chronic sleep disruption including insomnia would lead to ER as a result of diminished cognitive functioning by affecting frontal brain regions and diminishing control over executive functions (235–237). For example, people suffering from insomnia show dysfunctions in the neural circuitry underlying ER (238–240) and impairments in both basic cognitive functions and higher-order cognitive processing involved in supervisory control, problem solving, flexibility and self-control (241, 242). As such, insomnia is frequently seen in BD (243–246) and related to emotional hyper-reactivity during remitted phases (247, 248). Further, a cross-sectional study assessing the potential association between insomnia, ED and suicidality in subjects with BD found that insomnia symptoms significantly predicted the severity of depressive symptoms, ED, and suicidality in subjects with BD (69).

### Evening chronotype

Morningness-eveningness, or chronotype, reflects individual variations in diurnal preferences (249). The evening chronotype appears to be independently linked to ER, regardless of factors like sleep quality, duration, timing, or debt (250). For instance, individuals with a morning chronotype report fewer attentional and emotional difficulties, supported by evidence at the brain structural level (251). Additionally, research investigating the association between later chronotypes and amygdala reactivity to negative (e.g., fearful) facial expressions found that evening chronotypes exhibit heightened amygdala responses to negative stimuli and reduced dorsal ACC-amygdala functional connectivity (249). This suggests impaired ER circuitry independent of sleep quality, depression diagnosis, and family history (249). Additionally, research shows a negative bias in emotion processing in late chronotypes and increased difficulty in anger and sadness recognition for expressive suppressor morning-types, highlighting further the importance of chronotype as a relevant variable in ER and emotional functioning (252).

Studies consistently show that individuals with BD are more likely than healthy controls to display an evening chronotype, characterized by a preference for later sleep, wakefulness, and activity patterns (55, 225). This relationship is corroborated by findings using objective chronotype measures, such as actigraphy (253, 254). The evening chronotype is thought to have a genetic basis, as evidenced by heritability studies (255, 256). While disrupted circadian rhythms and evening chronotypes are commonly observed in BD, their specific roles in mood episodes or emotionality remain uncertain (55). However, chronotype has been proposed as a potential biomarker for lithium treatment response in BD (257, 258).

Lithium may exert its mood-stabilizing effects partially through modulating the circadian system (43), as alterations in circadian CLOCK genes have been implicated in lithium response (259, 260). Notably, lithium has been shown to extend the circadian period and enhance circadian rhythm amplitude (261). More specifically, lithium inhibits glycogen synthase kinase-3 beta, which phosphorylates and stabilizes the circadian nuclear receptor rev-erbalpha (262) and rev-erbalpha influences mood regulation through circadian control of the dopaminergic system (263, 264). These finding may suggest that targeting circadian mechanisms could be a key avenue for improving ED in BD.

## Clinical relevance of the findings

### Assessment

ER difficulties may be detected as part of a standard clinical psychiatric assessment. Specific tools such as the Difficulties in Emotion Regulation Scale (DERS) and the Affective Lability Scale (ALS) may also be used to assess emotional reactivity and poor emotional clarity at individual time points (265). However, digital tools such as ecological momentary assessment (EMA) allow real-time tracking of emotional states and therefore can provide insights into dynamic fluctuations that may be missed with traditional tools (266). Behavioural neuropsychological assessments, particularly those relating to executive function, can be used to further clarify cognitive impairments associated with emotional dysregulation (267). Neuroimaging and physiological measures like heart rate variability, may also potential as adjunctive methods for assessment, although these are not used widely in clinical practice (267).

### Treatments

Psychotherapeutic interventions, such as Dialectical Behaviour Therapy (DBT) and Cognitive Behavioral Therapy (CBT), teach skills such as mindfulness, distress tolerance, and cognitive restructuring that may help to support emotional regulation in patients with BD (268). Emotion-focused therapies, such as emotion regulation group therapy (ERGT), have specifically shown efficacy in reducing emotional reactivity, but this modality is less widely available (268).

Pharmacological treatments, including mood stabilizers like lithium and valproate, may be pivotal in managing ED. As discussed before, lithium, not only stabilises mood but also appears to modulate neural circuits involved in ER, including the prefrontal cortex and amygdala (134). It has been shown to reduce emotional lability, impulsivity, and aggression, providing long-term protection against mood destabilization (134). Likewiese, Valproate, a commonly-used mood stabiliser in BD, enhances gamma-aminobutyric acid (GABA) activity in the brain, which reduces hyperactivity in ER pathways (269), mitigating emotional reactivity and impulsivity. However, recent UK MHRA guidance on valproate is limiting its ongoing use in both males and females of reproductive age (MHRA 2024). Nonetheless, these medications, often used in combination with psychotherapeutic approaches, continue to play a critical role in improving ER and preventing relapse. Furthermore, emerging technologies, such as smartphone-based interventions and virtual reality platforms, show promise as novel supportive therapies to enhance ER assessment and management (269), which will be discussed further in detail in “Future Directions”.

In addition to the psychotherapeutic and pharmacological treatments, there is growing interest in the ketogenic diet as a treatment for BD (270, 271). This is due to BD being recognised more as a disorder of energy dysregulation (272) and mitochondrial dysfunction (59). However, although evidence for mitochondrial dysfunction in BD is well-established, the genetic association of mitochondria with BD remains weak (79). This implies that mitochondrial dysfunction may act as an intermediate phenotype shaped by other causal risk factors, including environmental influences, infections, and drug exposure, rather than being a direct result of genetic factors (79).

Ketogenic diet may improve metabolic and biochemical features of BD (271) such as reduction in oxidative stress and inflammation, improved mitochondrial function and biogenesis, improved glutamate/GABA transmission and reductions in intracellular sodium and calcium (273), increase of BDNF (274), reduction of BMI and control of obesity (275) and regulation of mood (276). Thus, the ketogenic diet presents a promising avenue for exploring dietary interventions as potential therapeutic strategies for BD, providing an opportunity to assess its effects within specific domains of psychopathology such as cognition and mood (270). A comprehensive understanding of how the ketogenic diet can ameliorate mitochondrial function, related gene expression, reward neural network, dopaminergic and GABAergic neurotransmission abnormalities in BD is also crucial to help guide personalized interventions and provide targets for novel therapies for BD (79).

## Future directions

### Digital phenotyping

Diagnosis in BD have centred on identifying episodes, with clinical outcomes often reduced to a binary approach (4). This approach is particularly problematic in BD, where mood can vary significantly, often in both directions, within any given evaluation period (4). A potential solution involves implementing more frequent and real-time mood assessments (e.g., ESM/EMA). Leveraging new technologies can enhance our ability to capture mood and mental state data with greater efficiency and accuracy, and it can push BD research beyond a sole focus on psychopathology by incorporating physiological, behavioural, and environmental data (4). For example, actigraphy has been used to derive objective measures of sleep characteristics (277, 278). An actigraphy study examining the interactive relationships among impulsivity, sleep disturbance, and circadian rhythm disturbance as predictors of mood symptoms in a high-risk and recent-onset BD sample found that less total actigraphic sleep time predicted more next-day depressive symptoms (220). Longer total sleep time was associated with fewer next-day hypomanic symptoms when participants had low, but not high, behavioural impulsivity. Additionally, for participants with high self-reported impulsivity, later dim light melatonin onset time was associated with higher next-day depressive symptoms. Another study looking at rest-activity cycles in euthymic BD patients found patients who presented with unstable rest-activity cycles had delayed sleep-wake timing and greater mood variability, and younger age (279). Lastly, patients who had later/irregular rest-activity patterns had greater lifetime manic-hypomanic and depressive symptoms (228).

Actigraphy has also been used in 24-hour patterns of motor activity (280), sedentary behaviour (281), and physical activity or exercise (282). A case-control study of 242 patients with BD found bidirectional associations between motor activity with subjectively rated energy level and sleep duration as well as unidirectional associations between motor activity and mood level (283). Further, a systematic review looking at motor activity patterns in patients with BD and MDD found lower mean activity (284, 285). In addition, patients with BD and MDD with lower amplitude of the 24-h rest-activity rhythm report greater mood instability (286).

Widespread availability of connected and wearable devices (e.g., smartphones) had led to digital phenotyping based on high-resolution measurement of remotely captured data streams (287). Given that the primary characteristic of BD is a biphasic energy shift, monitoring the corresponding phasic dysregulation in mood, sleep, and behaviour is gaining attention (288). This comprehensive approach may uncover biological correlates and, ultimately, the underlying mechanisms of the disorder, such as ED. Digital technologies for example in metabolomics, with further exploration, may also assist in moving towards more personalised and stratified healthcare approaches (289).

### Measurement issues

Previous studies using subjective measures of ER have generally not identified any abnormalities in patients with BD (35, 266, 290, 291). This may be due to the limited sensitivity of the self- report measures used (292). While neuroimaging is a sensitive method for detecting subtle neural changes during ER, it is not practical for diagnostic evaluations in routine clinical practice (292). Therefore, there is a need for research into improved, cost-effective measures with sensitivity to detect abnormalities in the reactivity to and regulation of emotions in BD to use in diagnostic evaluations in routine clinical practice (292). A multidisciplinary approach combining subtle behavioural measures (e.g., eye-tracking and facial expressions) with subjective responses during emotion reactivity and regulation may offer a more sensitive and cost-effective alternative for detecting aberrant ER in BD (292). Supporting this, a study investigating trait-related abnormalities in emotional reactivity and regulation in BD using novel behavioural measures, such as facial expressions and eye movements, found subtle differences in visual gaze patterns and facial displays of emotion between remitted BD patients and healthy controls during emotion processing and regulation (292). The authors suggest that these subtle differences in visual gaze and facial expressions may provide sensitivity comparable to neuroimaging techniques, while being less costly and more feasible for clinical applications. Further, evidence from fMRI studies is often inconsistent (60). On the contrary, studies using eye‐tracking methodology revealed relatively consistent abnormalities such as oculomotor abnormalities including slower inhibitory control during facial emotion processing in remitted and symptomatic patients (293–295). The consistency within eye-tracking studies may suggest that this may be a sensitive measure to detect subtle behavioural differences in ER, particularly implicit ER (60).

### The need for longitudinal data

To determine whether an etiological mechanism precedes the development of ED in BD, the biomarkers should be assessed before the development of BD. Thus, prospective studies investigating biomarkers underlying ED in BD before the onset of the first episode are necessary. This is crucial also because there is a lack of reliable disease biomarkers in BD (296, 297) and this is partly due to the cross-sectional nature of the biomarker studies (298). The results can help develop novel treatments (e.g., preventive emotion-based treatments) that aim to improve emotion resilience in at-risk individuals by enhancing their emotional knowledge, and regulation which in return delay the onset of the disorder or reduce the severity of pathological affective states and ED (29, 299). The identification of specific endophenotypes (e.g., aberrant ER and emotion and reward processing) that reflect underlying pathophysiological processes could later be implemented in the clinical assessments to improve future diagnostic discrimination between UD and BD (29). However, despite the need for large cohort studies, obtaining funding has been a hurdle (300). Many cohort studies (e.g., Iowa 500, STEP- BD, EMBLEM, WAVE-BD, the Stanley Foundation Network, FACE-BD, CIBERSAM) were limited in time due to short-term funding, missing the opportunity of following the participants over a long time (300). Therefore, the creation of an international early BD data network could coordinate international big data approaches and integrate with real-world clinical interventions (298). In addition to the promising “Big Data” and machine learning approaches, there is also a need for an overarching theoretical framework (301). Recently, Mansur et al. (301) proposed a comprehensive disease model of BD. According to the model, disruptions in the network that regulates energy homeostasis, within its paths and/or nodes, leads to “BD-like phenomena” and a “BD-like clinical trajectory”, applying the equifinality principle and the “homeostatic property cluster” concept (301). This disease model could also be tested longitudinally in its relation to ED in BD as research showed that subjective mood in BD is directly affected by subjective energy and objectively measured activity (283).

## Conclusions

ED is a transdiagnostic construct observed in individuals with BD and is significantly correlated with its symptomatology (40). While ED is recognized as a transdiagnostic dimension and a potential target for personalized interventions, it is essential to delineate the aspects of ED that are specific to BD versus those shared with other psychiatric disorders (13). Various biological mechanisms, including altered structural and functional brain alterations, inflammation, HPA axis, dysfunction, genetics and circadian rhythm disruptions, have been linked to ED in BD. Despite this, ER has often been studied independently of these biological underpinnings. Given the strong evidence connecting biological mechanisms to ER difficulties in BD, adopting a more integrative perspective is both timely and necessary.

If robust evidence supports the hypothesis that biological mechanisms underpin emotional difficulties in BD, it could offer a novel framework for understanding and treating these challenges (25). However, no biomarker for BD has yet demonstrated sufficient robustness, specificity, or clinical utility for diagnostic or therapeutic use (302). This gap in understanding the pathophysiology of BD and the lack of reliable biomarkers have constrained clinical practice to a trial-and-error approach (303). To address these limitations, larger-scale and more comprehensive studies are needed to generate conclusive results and identify potential clinico-pathological correlates and subgroups within BD (302). Longitudinal, international cohort studies with repeated multiple biomarkers that integrate peripheral blood measures, electrophysiology, neuroimaging and cognitive neuroscience, coupled by deep clinical phenotyping may overcome current methodological problems in biomarker studies in psychiatry (296).

Integrating advanced biomarker research with investigations into emotional processes in BD could promote a more holistic understanding of functionality and vulnerability, ultimately informing novel treatment interventions (25). Such research may also facilitate the development of a new biological model of emotion in BD, focusing on the interaction of various biological systems involved in both voluntary and automatic processes of ER. This model could not only elucidate the biological basis of ER in BD but also be extended to youth at risk for BD, enabling earlier interventions and preventive strategies. Furthermore, exploring how ER difficulties respond to targeted biological interventions, such as treatments addressing inflammation or cognitive impairments, could clarify the directional relationship between biological factors and ER in BD. This knowledge has the potential to significantly advance the understanding and treatment of ED in BD (25).

## References

[1] McIntyre RS Alda M Baldessarini RJ Bauer M Berk M Correll CU. The clinical characterization of the adult patient with bipolar disorder aimed at personalization of management. World Psychiatry. (2022) 21:364–87. 10.1002/wps.20997 PMC9453915 36073706

[2] Yatham LN Kauer-Sant’Anna M Bond DJ Lam RW Berk M. Innovation in bipolar disorder treatment. Nat Rev Drug Discovery. (2018) 17:387–405.

[3] McKnight RF Bilderbeck AC Miklowitz DJ Hinds C Goodwin GM Geddes JR. Longitudinal mood monitoring in bipolar disorder: course of illness as revealed through a short messaging service. J Affect Disord. (2017) 223:139–45. 10.1016/j.jad.2017.07.029 28753472

[4] Harrison PJ Cipriani A Harmer CJ Nobre AC Saunders K Goodwin GM. Innovative approaches to bipolar disorder and its treatment. Ann New York Acad Sci. (2016) 1366:76–89. 27111134 10.1111/nyas.13048 PMC4850752

[5] Mason L Eldar E Rutledge RB. Mood instability and reward dysregulation—a neurocomputational model of bipolar disorder. JAMA Psychiatry. (2017) 74:1275–6. 10.1001/jamapsychiatry.2017.3163 29049438

[6] Beauchaine TP Cicchetti D. Emotion dysregulation and emerging psychopathology: A transdiagnostic, transdisciplinary perspective. Dev Psychopathol. (2019) 31:799–804. doi: 10.1017/S0954579419000671 31290735

[7] Broome MR He Z Iftikhar M Eyden J Marwaha S. Neurobiological and behavioural studies of affective instability in clinical populations: a systematic review. Neurosci Biobehav Rev. (2015) 51:243–54. doi: 10.1016/j.neubiorev.2015.01.021 25662294

[8] Marwaha S He Z Broome M Singh SP Scott J Eyden J. How is affective instability defined and measured? A systematic review. psychol Med. (2014) 44:1793–808. doi: 10.1017/S0033291713002407 24074230

[9] Marwaha S Price C Scott J Weich S Cairns A Dale J. Affective instability in those with and without mental disorders: a case control study. J Affect Disord. (2018) 241:492–8. doi: 10.1016/j.jad.2018.08.046 30149337

[10] D’Agostino A Covanti S Rossi Monti M Starcevic V. Reconsidering emotion dysregulation. Psychiatr Q. (2017) 88:807–25. doi: 10.1007/s11126-017-9499-6 28194549

[11] Thompson RA. Emotion dysregulation: A theme in search of definition. Dev Psychopathol. (2019) 31:805–15. doi: 10.1017/S0954579419000282 31030684

[12] Pozzi E Vijayakumar N Rakesh D Whittle S. Neural correlates of emotion regulation in adolescents and emerging adults: A meta-analytic study. Biol Psychiatry. (2021) 89:194–204. doi: 10.1016/j.biopsych.2020.08.006 33268030

[13] De Prisco M Villani A Della Vecchia A Gaudiano C Cigliano G Cecere A. Emotion dysregulation in bipolar disorder compared to other mental illnesses: a systematic review and meta-analysis. psychol Med. (2023) 53:7484–503. doi: 10.1017/S003329172300243X PMC10951413 37842774

[14] Van Rheenen TE Murray G Rossell SL. Emotion regulation in bipolar disorder: profile and utility in predicting trait mania and depression propensity. Psychiatry Res. (2015) 225:425–32. doi: 10.1016/j.psychres.2014.12.001 25537486

[15] Artiach Hortelano P Martens MA Pringle A Harmer CJ. Effect of lithium administration on brain activity under an emotion regulation paradigm in healthy participants: a functional magnetic resonance imaging study. Psychopharmacology. (2023) 240:1719–34. doi: 10.1007/s00213-023-06395-7 PMC10349753 37338568

[16] Brady RO Masters GA Mathew IT Margolis A Cohen B Keshavan MS. State dependent cortico-amygdala circuit dysfunction in bipolar disorder. J Affect Disord. (2016) 201:79–87. doi: 10.1016/j.jad.2016.04.052 27177299 PMC5087105

[17] Johnson SL Tharp JA Peckham AD McMaster KJ. Emotion in bipolar I disorder: Implications for functional and symptom outcomes. J Abnormal Psychol. (2016) 125:40. doi: 10.1037/abn0000116 PMC4701591 26480234

[18] Li L Ji E Tang F Magioncalda P Martino M Mathalon DH. Abnormal brain activation during emo- tion processing of euthymic bipolar patients taking different mood stabilizers. Brain Imag Behav. (2019) 13:905–13. doi: 10.1007/s11682-018-9915-z 29909585

[19] Faurholt-Jepsen M Frost M Busk J Christensen EM Bardram JE Vinberg M. Is smartphone-based mood instability associated with stress, quality of life, and functioning in bipolar disorder? Bipolar Disord. (2019) 21:611–20. 10.1111/bdi.12796 31081991

[20] Faurholt-Jepsen M Busk J Bardram JE Stanislaus S Frost M Christensen EM. Mood instability and activity/energy instability in patients with bipolar disorder according to day-to-day smartphone-based data–An exploratory *post hoc* study. J Affect Disord. (2023) 334:83–91. doi: 10.1016/j.jad.2023.04.139 37149047

[21] Rucklidge JJ. Psychosocial functioning of adolescents with and without paediatric bipolar disorder. J Affect. Disord. (2006) 91:181–8. 10.1016/j.jad.2006.01.001 16478633

[22] Ulusoy SI Gülseren Ş.A Özkan N Bilen C. Facial emotion recognition deficits in patients with bipolar disorder and their healthy par- ents. Gen Hosp Psychiatry. (2020) 65:9–14. doi: 10.1016/j.genhosppsych.2020.04.008 32361661

[23] Muhtadie L Johnson SL Carver CS Gotlib IH Ketter TA. A profile approach to impulsivity in bipolar disorder: The key role of strong emotions. Acta Psychiatrica Scandinavica. (2014) 129:100–8. doi: 10.1111/acps.12136 PMC4346162 23600731

[24] Gruber J Harvey AG Gross JJ. When trying is not enough: Emotion regulation and the effort–success gap in bipolar disorder. Emotion. (2012) 12:997. doi: 10.1037/a0026822 22251049

[25] Lima IM Peckham AD Johnson SL. Cognitive deficits in bipolar disorders: Implications for emotion. Clin Psychol Rev. (2018) 59:126–36. doi: 10.1016/j.cpr.2017.11.006 PMC6404979 29195773

[26] Johnson SL Carver CS. Emotion-relevant impulsivity predicts sustained anger and aggression after remission in bipolar I disorder. J Affect Disord. (2016) 189:169–75. doi: 10.1016/j.jad.2015.07.050 26437231

[27] Johnson SL Carver CS Tharp JA. Suicidality in bipolar disorder: The role of emotion-triggered impulsivity. Suicide Life-threatening Behav. (2017) 47:177–92. doi: 10.1111/sltb.12274 PMC5788807 27406282

[28] Kjærstad HL Mistarz N Coello K Stanislaus S Melbye SA Harmer CJ. Aberrant cognition in newly diagnosed patients with bipolar disorder and their unaffected relatives. psychol Med. (2020) 50:1808–19. doi: 10.1017/S0033291719001867 31456531

[29] Miskowiak KW Kjaerstad HL Meluken I Petersen JZ Maciel BR Kohler CA. The search for neuroimaging and cognitive endophenotypes: a critical systematic review of studies involving unaffected first-degree relatives of individuals with bipolar disorder. Neurosci Biobehav Rev. (2017) 73:1–22. doi: 10.1016/j.neubiorev.2016.12.011 27979650

[30] Birmaher B Goldstein BI Axelson DA Monk K Hickey MB Fan J. Mood lability among offspring of parents with bipolar disorder and community controls. Bipolar Disord. (2013) 15:253–63. doi: 10.1111/bdi.2013.15.issue-3 PMC3644367 23551755

[31] Kessing LV Miskowiak K. Does cognitive dysfunction in bipolar disorder qualify as a diagnostic intermediate phenotype? – A perspective paper. Front Psychiatry. (2018) 9:1–7. doi: 10.3389/fpsyt.2018.00490 30349492 PMC6186783

[32] de Almeida JRC Phillips ML. Distinguishing between unipolar depression and bipolar depression: current and future clinical and neuroimaging perspectives. Biol Psychiatry. (2013) 73:111–8. doi: 10.1016/j.biopsych.2012.06.010 PMC3494754 22784485

[33] Phillips ML Ladouceur CD Drevets WC. A neural model of voluntary and automatic emotion regulation: implications for understanding the pathophysiology and neurodevelopment of bipolar disorder. Mol Psychiatry. (2008) 13:833–57. doi: 10.1038/mp.2008.65 PMC2745893 18574483

[34] Townsend JD Torrisi SJ Lieberman MD Sugar CA Bookheimer SY Altshuler LL. Frontal-amygdala connectivity alterations during emotion downregulation in bipolar I disorder. Biological Psychiatry. (2013) 73(2):127–35. 10.1016/j.biopsych.2012.06.030 PMC3525751 22858151

[35] Kanske P Schönfelder S Forneck J Wessa M. Impaired regulation of emotion: neural correlates of reappraisal and distraction in bipolar disorder and unaffected relatives. Trans Psychiatry. (2015) 5:e497–7. doi: 10.1038/tp.2014.137 PMC4312831 25603413

[36] Kjærstad HL de Siqueira Rotenberg L Knudsen GM Vinberg M Kessing LV Macoveanu J. The longitudinal trajectory of emotion regulation and associated neural activity in patients with bipolar disorder: a prospective fMRI study. Acta Psychiatrica Scandinavica. (2022) 146:568–82. 10.1111/acps.13488 PMC9804505 36054343

[37] Mesbah R Koenders MA van der Wee NJ Giltay EJ van Hemert AM de Leeuw M. Association between the fronto-limbic network and cognitive and emotional functioning in individuals with bipolar disorder: a systematic review and meta-analysis. JAMA Psychiatry. (2023) 80:432–40. doi: 10.1001/jamapsychiatry.2023.0131 PMC10061312 36988918

[38] Townsend J Altshuler LL. Emotion processing and regulation in bipolar disorder: a review. Bipolar Disord. (2012) 14:326–39. doi: 10.1111/j.1399-5618.2012.01021.x 22631618

[39] Dodd A Lockwood E Mansell W Palmier-Claus J. Emotion regulation strategies in bipolar disorder: A systematic and critical review. J Affect Disord. (2019) 246:262–84. doi: 10.1016/j.jad.2018.12.026 30590289

[40] Oliva V De Prisco M Fico G Possidente C Fortea L Montejo L. Correlation between emotion dysregulation and mood symptoms of bipolar disorder: A systematic review and meta-analysis. Acta Psychiatrica Scandinavica. (2023) 148:472–90. doi: 10.1111/acps.v148.6 37740499

[41] Leucht S van Os J Jäger M Davis JM. Prioritization of psychopathological symptoms and clinical characterization in psychiatric diagnoses: A narrative review. JAMA Psychiatry. (2024). doi: 10.1001/jamapsychiatry.2024.2652 39259534

[42] Uher R Zwicker A. Etiology in psychiatry: embracing the reality of poly-gene-environmental causation of mental illness. World Psychiatry. (2017) 16:121–9. doi: 10.1002/wps.20436 PMC5428165 28498595

[43] Carpenter JS Crouse JJ Scott EM Naismith SL Wilson C Scott J. Circadian depression: a mood disorder phenotype. Neurosci Biobehav Rev. (2021) 126:79–101. doi: 10.1016/j.neubiorev.2021.02.045 33689801

[44] Bigot M Alonso M Houenou J Sarrazin S Dargél AA Lledo PM. An emotional-response model of bipolar disorders integrating recent findings on amygdala circuits. Neurosci Biobehav Rev. (2020) 118:358–66. doi: 10.1016/j.neubiorev.2020.07.037 32739421

[45] Kjærstad HL Søhol K Vinberg M Kessing LV Miskowiak KW. The trajectory of emotional and non-emotional cognitive function in newly diagnosed patients with bipolar disorder and their unaffected relatives: A 16-month follow-up study. Eur Neuropsychopharmacol. (2023) 67:4–21. doi: 10.1016/j.euroneuro.2022.11.004 36462414

[46] McIntyre RS Berk M Brietzke E Goldstein BI López-Jaramillo C Kessing LV. Bipolar disorders. Lancet. (2020) 396:1841–56. doi: 10.1016/S0140-6736(20)31544-0 33278937

[47] Young AH Juruena MF. The neurobiology of bipolar disorder. In: Bipolar disorder: from neuroscience to treatment (2021) Cham, Switzerland: Springer. p. 1–20. 10.1007/7854_2020_179 33442840

[48] Rowland T Perry BI Upthegrove R Barnes N Chatterjee J Gallacher D. Neurotrophins, cytokines, oxidative stress mediators and mood state in bipolar disorder: systematic review and meta-analyses. Br J Psychiatry. (2018) 213:514–25. doi: 10.1192/bjp.2018.144 PMC6429261 30113291

[49] Hibar DP Westlye LT Doan NT Jahanshad N Cheung JW Ching CRK. Cortical abnormalities in bipolar disor- der: an MRI analysis of 6503 individuals from the ENIGMA Bipolar Disorder Working Group. Mol Psychiatry. (2017) 22:1231–9. 10.1038/mp.2017.73 PMC5668195 28461699

[50] Phillips ML Swartz HA. A critical appraisal of neuroimaging studies of bipolar disorder: toward a new concep- tualization of underlying neural circuitry and a road map for future research. Am J Psychiatry. (2014) 171:829–43. doi: 10.1176/appi.ajp.2014.13081008 PMC4119497 24626773

[51] Wise T Radua J Via E Cardoner N Abe O Adams TM. Voxel-based meta-analytical evidence of structural disconnectivity in major depression and bipolar disorder. Biol Psychiatry. (2016) 79:293–302. doi: 10.1016/j.biopsych.2015.03.004 25891219

[52] Brown NC Andreazza AC Young LT. An updated meta-analysis of oxidative stress markers in bipolar disorder. Psychiatry Research. (2014) 218(1–2):61–68. 10.1016/j.psychres.2014.04.005 24794031

[53] Andreazza AC Kato T Young LT Yatham LN Kapczinski F Shao L. Bipolar disorder as a mitochondrial disease. Biol Psychiatry. (2017) 81:2017. 10.1016/j.biopsych.2017.09.018 29050637

[54] Goldsmith DR Rapaport MH Miller BJ Ellman LM Robinson DG Addington J. A meta-analysis of blood cytokine network alterations in psychiatric patients: comparisons between schizophrenia, bipolar disorder and depression. Mol Psychiatry. (2016) 21:1696–709. doi: 10.1038/mp.2016.3 PMC6056174 26903267

[55] Melo MCA Abreu RLC Linhares Neto VB de Bruin PFC de Bruin VMS. Chronotype and circadian rhythm in bipolar disorder: a systematic review. Sleep Med Rev. (2017) 34:46–58. doi: 10.1016/j.smrv.2016.06.007 27524206

[56] Ashok AH Marques TR Jauhar S Nour MM Goodwin GM Young AH. The dopamine hypothesis of bipolar affective disorder: the state of the art and implications for treatment. Mol Psychiatry. (2017) 22:666–79. doi: 10.1038/mp.2017.16 PMC5401767 28289283

[57] Anderson G Maes M. Bipolar disorder: role of immune-inflammatory cytokines, oxidative and nitrosative stress and tryptophan catabolites. Curr Psychiatry Rep. (2015) 17:1–9. doi: 10.1007/s11920-014-0541-1 25620790

[58] Andreazza AC Young LT. The neurobiology of bipolar disorder: identifying targets for specific agents and synergies for combination treatment. Int J Neuropsychopharmacol. (2014) 17:1039–52. doi: 10.1017/S1461145713000096 23449044

[59] Berk M Kapczinski F Andreazza AC Dean OM Giorlando F Maes M. Pathways underlying neuroprogression in bipolar disorder: focus on inflammation, oxidative stress and neurotrophic factors. Neurosci Biobehav Rev. (2011) 35:804–17. doi: 10.1016/j.neubiorev.2010.10.001 20934453

[60] Miskowiak KW Seeberg I Kjaerstad HL Burdick KE Martinez-Aran A del Mar Bonnin C. Affective cognition in bipolar disorder: a systematic review by the ISBD targeting cognition task force. Bipolar Disord. (2019) 21:686–719. doi: 10.1111/bdi.12834 31491048

[61] Van Rheenen TE Ganella EP Bauer IE Bartholomeusz CF. Characterization of social cognitive deficits on the schizophrenia- bipolar disorder spectrum: an overview of current evidence. In: Lewandowski KE Moustafa AA, editors. Social cognition in psychosis, 1 ed. Elsevier, Cambridge, MA (2019).

[62] Cotter J Barnett JH. Using affective cognition to enhance precision psychiatry. Front Psychiatry. (2018) 9:288. doi: 10.3389/fpsyt.2018.00288 30008680 PMC6033989

[63] Powers A Almli L Smith A Lori A Leveille J Ressler KJ. A genome-wide association study of emotion dysregulation: Evidence for interleukin 2 receptor alpha. J Psychiatr Res. (2016) 83:195–202. doi: 10.1016/j.jpsychires.2016.09.006 27643478 PMC5896292

[64] Tseng CEJ Gilbert TM Catanese MC Hightower BG Peters AT Parmar AJ. *In vivo* human brain expression of histone deacetylases in bipolar disorder. Trans Psychiatry. (2020) 10:224. doi: 10.1038/s41398-020-00911-5 PMC7343804 32641695

[65] Saccaro LF Crokaert J Perroud N Piguet C. Structural and functional MRI correlates of inflammation in bipolar disorder: A systematic review. J Affect Disord. (2023) 325:83–92. doi: 10.1016/j.jad.2022.12.162 36621677

[66] Koenders MA Dodd AL Karl A Green MJ Elzinga BM Wright K. Understanding bipolar disorder within a biopsychosocial emotion dysregulation framework. J Affect Disord Rep. (2020) 2:100031. doi: 10.1016/j.jadr.2020.100031

[67] Hosang GM Uher R Keers R Cohen-Woods S Craig I Korszun A. Stressful life events and the brain-derived neurotrophic factor gene in bipolar disorder. J Affect Disord. (2010) 125:345–9. doi: 10.1016/j.jad.2010.01.071 20172611

[68] Alloy LB Ng TH Titone MK Boland EM. Circadian rhythm dysregulation in bipolar spectrum disorders. Curr Psychiatry Rep. (2017) 19:1–10. doi: 10.1007/s11920-017-0772-z 28321642 PMC6661150

[69] Palagini L Cipollone G Masci I Caruso D Paolilli F Perugi G. Insomnia symptoms predict emotional dysregulation, impulsivity and suicidality in depressive bipolar II patients with mixed features. Compr Psychiatry. (2019) 89:46–51. doi: 10.1016/j.comppsych.2018.12.009 30593973

[70] Gordovez FJA McMahon FJ. The genetics of bipolar disorder. Mol Psychiatry. (2020) 25:544–59. doi: 10.1038/s41380-019-0634-7 31907381

[71] Hayashi A Le Gal K Södersten K Vizlin-Hodzic D Ågren H Funa K. Calcium-dependent intracellular signal pathways in primary cultured adipocytes and ANK3 gene variation in patients with bipolar disorder and healthy controls. Mol Psychiatry. (2015) 20:931–40. doi: 10.1038/mp.2014.104 PMC4759096 25311363

[72] Lim CH Zain SM Reynolds GP Zain MA Roffeei SN Zainal NZ. Genetic association of LMAN2L gene in schizophrenia and bipolar disorder and its interaction with ANK3 gene polymorphism. Progress in Neuro-Psychopharmacology and Biological Psychiatry. (2014) 54:157–162. 10.1016/j.pnpbp.2014.05.017 24914473

[73] Lippard ETC Jensen KP Wang F Johnston JAY Spencer L Pittman B. Effect of ANK3 variation on gray and white matter in bipolar disorder. Mol Psychiatry. (2017) 22:1345–51. doi: 10.1038/mp.2016.76 PMC5133179 27240527

[74] Ferreira MAR O’Donovan MC Meng YA Jones IR Ruderfer DM Jones L. Collaborative genome-wide association analysis supports a role for ANK3 and CACNA1C in bipolar disorder. Nat Genet. (2008) 40:1056–8. doi: 10.1038/ng.209 PMC2703780 18711365

[75] Stahl EA Breen G Forstner AJ McQuillin A Ripke S Trubetskoy V. Genome-wide association study identifies 30 loci associated with bipolar disorder. Nat Genet. (2019) 51:793–803. doi: 10.1038/s41588-019-0397-8 31043756 PMC6956732

[76] Ikeda M Takahashi A Kamatani Y Okahisa Y Kunugi H Mori N. A genome-wide association study identifies two novel susceptibility loci and trans population polygenicity associated with bipolar disorder. Mol Psychiatry. (2018) 23:639–47. doi: 10.1038/mp.2016.259 PMC5822448 28115744

[77] Jiang X Detera-Wadleigh SD Akula N Mallon BS Hou L Xiao T. Sodium valproate rescues expression of TRANK1 in iPSC-derived neural cells that carry a genetic variant associated with serious mental illness. Mol Psychiatry. (2019) 24:613–24. doi: 10.1038/s41380-018-0207-1 PMC6894932 30135510

[78] Mathieu F Etain B Dizier MH Lajnef M Lathrop M Cabon C. Genetics of emotional reactivity in bipolar disorders. J Affect Disord. (2015) 188:101–6. doi: 10.1016/j.jad.2015.08.037 26349599

[79] Freyberg Z Andreazza AC McClung CA Phillips ML. Linking mitochondrial dysfunction, neurotransmitter, neural network abnormalities and mania: Elucidating neurobiological mechanisms of the therapeutic effect of the ketogenic diet in Bipolar Disorder. Biol Psychiatry: Cogn Neurosci Neuroimaging. (2024). 10.1016/j.bpsc.2024.07.011 PMC11754533 39053576

[80] Ludwig B Dwivedi Y. Dissecting bipolar disorder complexity through epigenomic approach. Mol Psychiatry. (2016) 21:1490–8. 10.1038/mp.2016.123 PMC5071130 27480490

[81] MaChado-Vieira R Ibrahim L Zarate CA Jr. Histone deacety- lases and mood disorders: Epigenetic programming in gene- environment interactions. CNS Neurosci Ther. (2011) 17:699–704. 20961400 10.1111/j.1755-5949.2010.00203.x PMC3026916

[82] Ryu H Lee J Olofsson BA Mwidau A Dedeoglu A Escudero M. Histone deacetylase inhibitors prevent oxidative neuronal death independent of expanded polyglutamine repeats via an Sp1-dependent pathway. Proc Natl Acad Sci U.S.A. (2003) 100:4281–4286. 12640146 10.1073/pnas.0737363100 PMC153084

[83] Xiang B Liu K Yu M Liang X Zhang J Lei W. Systematic genetic analyses of genome-wide association studydata reveal an association between the key nucleosome remodeling anddeacetylase complex and bipolar disorder development. Bipolar Disord. (2018) 20:370–80. doi: 10.1111/bdi.2018.20.issue-4 29280245

[84] Pisanu C Papadima EM Del Zompo M Squassina A. Understanding the molecular mechanisms underlying mood stabilizer treatments in bipolar disorder: potential involvement of epigenetics. Neurosci Lett. (2018) 669:24–31. doi: 10.1016/j.neulet.2016.06.045 27343410

[85] Delcuve GP Khan DH Davie JR. Roles of histone deacetylases in epigenetic regulation: emerging paradigms from studies with inhibitors. Clin Epigenetics. (2012) 4:5. doi: 10.1186/1868-7083-4-5 22414492 PMC3320549

[86] Mitchell CP Chen Y Kundakovic M. Histone deacetylase inhibitors decrease reelin promoter methylation *in vitro* . J Neurochem. (2005) 93:483–92. doi: 10.1111/j.1471-4159.2005.03040.x 15816871

[87] Phiel CJ Zhang F Huang EY Guenther MG Lazar MA Klein PS. Histone deacetylase is a direct target of valproic acid, a potentanticonvulsant, mood stabilizer, and teratogen. J.Biol.Chem. (2001) 276:36734–41. 10.1074/jbc.M101287200 11473107

[88] Wu S Zheng S-D Huang H-L Yan L-C Yin X-F Xu H-N. Lithium down-regulates histone deacetylase 1 (HDAC1) andinduces degradation of mutant huntingtin. J Biol Chem. (2013) 288:35604–16. doi: 10.1074/jbc.M113.479865 PMC3853296 24165128

[89] Leng Y Fessler EB Chuang DM. Neuroprotective effects of the mood stabilizer lamotrigine against glutamate excitotoxicity: Roles of chromatinremodelling and Bcl-2 induction. Int J Neuropsychopharmacol. (2013) 16:607–20. doi: 10.1017/S1461145712000429 PMC6324934 22564541

[90] Gyurak A Gross JJ Etkin A. Explicit and implicit emotion regulation: A dual-process framework. Cogn Emotion. (2011) 25:400–12. doi: 10.1080/02699931.2010.544160 PMC3280343 21432682

[91] Park C Rosenblat JD Lee Y Pan Z Cao B Iacobucci M. The neural systems of emotion regulation and abnormalities in major depressive disorder. Behav Brain Res. (2019) 367:181–8. doi: 10.1016/j.bbr.2019.04.002 30951753

[92] Ahmed YB Al-Bzour AN Alzghoul SM Ibrahim RB Al-Khalili AA Ghayda’a N. Limbic and cortical regions as functional biomarkers associated with emotion regulation in bipolar disorder: A meta-analysis of neuroimaging studies. J Affect Disord. (2023) 323:506–13. doi: 10.1016/j.jad.2022.11.071 36462610

[93] Zhang S Wang Y Deng F Zhong S Chen L Luo X. Disruption of superficial white matter in the emotion regulation network in bipolar disorder. NeuroImage Clin. (2018) 20:875–82. doi: 10.1016/j.nicl.2018.09.024 PMC6169099 30286386

[94] Linke JO Stavish C Adleman NE Sarlls J Towbin KE Leibenluft E. White matter microstructure in youth with and at risk for bipolar disorder. Bipolar Disord. (2020) 22:163–73. doi: 10.1111/bdi.12885 PMC7155105 31883419

[95] Acuff HE Versace A Bertocci MA Ladouceur CD Hanford LC Manelis A. Association of neuroimaging measures of emotion processing and regulation neural circuitries with symptoms of bipolar disorder in offspring at risk for bipolar disorder. JAMA Psychiatry. (2018) 75:1241–51. doi: 10.1001/jamapsychiatry.2018.2318 PMC6528787 30193355

[96] Kurtz M Mohring P Förster K Bauer M Kanske P. Deficits in explicit emotion regulation in bipolar disorder: a systematic review. Int J bipolar Disord. (2021) 9:1–23. doi: 10.1186/s40345-021-00221-9 33937951 PMC8089068

[97] Kebets V Favre P Houenou J Polosan M Perroud N Aubry JM. Fronto-limbic neural variability as a transdiagnostic correlate of emotion dysregulation. Trans Psychiatry. (2021) 11:545. doi: 10.1038/s41398-021-01666-3 PMC8530999 34675186

[98] McTeague LM Rosenberg BM Lopez JW Carreon DM Huemer J Jiang Y. Identification of common neural circuit disruptions in emotional processing across psychiatric disorders. Am J Psychiatry. (2020) 177:411–21. doi: 10.1176/appi.ajp.2019.18111271 PMC7280468 31964160

[99] Rolls ET. A non-reward attractor theory of depression. Neuroscience & Biobehavioral Reviews. (2016) 68:47–58. 10.1016/j.neubiorev.2016.05.007 27181908

[100] Nolen-Hoeksema S Watkins ER. A heuristic for developing transdiagnostic models of psychopathology: explaining multifinality and divergent trajectories. Perspect Psychol Sci. (2011) 6:589–609. doi: 10.1177/1745691611419672 26168379

[101] Motzkin JC Philippi CL Wolf RC Baskaya MK Koenigs M. Ventromedial prefrontal cortex is critical for the regulation of amygdala activity in humans. Biol Psychiatry. (2015) 77:276–84. doi: 10.1016/j.biopsych.2014.02.014 PMC4145052 24673881

[102] Myers-Schulz B Koenigs M. Functional anatomy of ventromedial prefrontal cortex: implications for mood and anxiety disorders. Mol Psychiatry. (2012) 17:132–41. doi: 10.1038/mp.2011.88 PMC3937071 21788943

[103] Hiser J Koenigs M. The multifaceted role of the ventromedial prefrontal cortex in emotion, decision making, social cognition, and psychopathology. Biol Psychiatry. (2018) 83:638–47. doi: 10.1016/j.biopsych.2017.10.030 PMC5862740 29275839

[104] Marin MF Zsido RG Song H. et al: Skin conductance responses and neural activations during fear conditioning and extinction recall across anxiety disorders. JAMA Psychiatry. (2017) 74:622–31. doi: 10.1001/jamapsychiatry.2017.0329 PMC5539837 28403387

[105] Richter A Petrovic A Diekhof EK. et al: Hyperresponsivity and impaired prefrontal control of the mesolimbic reward system in schizophrenia. J Psychiatr Res. (2015) 71:8–15. doi: 10.1016/j.jpsychires.2015.09.005 26522867

[106] Rai S Griffiths KR Breukelaar IA Barreiros AR Chen W Boyce P. Default-mode and fronto-parietal network connectivity during rest distinguishes asymptomatic patients with bipolar disorder and major depressive disorder. Trans Psychiatry. (2021) 11:547. doi: 10.1038/s41398-021-01660-9 PMC8542033 34689161

[107] Breukelaar IA Erlinger M Harris A Boyce P Hazell P Grieve SM. Investigating the neural basis of cognitive control dysfunction in mood disorders. Bipolar Disord. (2020) 22:286–95. doi: 10.1111/bdi.12844 31604366

[108] Han KM De Berardis D Fornaro M Kim YK. Differentiating between bipolar and unipolar depression in functional and structural MRI studies. Prog Neuropsychopharmacol Biol Psychiatry. (2019) 91:20–7. doi: 10.1016/j.pnpbp.2018.03.022 29601896

[109] Rive MM Mocking RJ Koeter MW van Wingen G de Wit SJ van den Heuvel OA. State-dependent differences in emotion regulation between unmedicated bipolar disorder and major depressive disorder. JAMA Psychiatry. (2015) 72:687–96. doi: 10.1001/jamapsychiatry.2015.0161 25945779

[110] Saccaro LF Schilliger Z Dayer A Perroud N Piguet C. Inflammation, anxiety, and stress in bipolar disorder and borderline personality disorder: A narrative review. Neurosci Biobehav Rev. (2021) 127:184–92. doi: 10.1016/j.neubiorev.2021.04.017 33930472

[111] Baczkowski B van Zutphen L Siep N Jacob GA Domes G Maier S. Deficient amygdala–prefrontal intrinsic connectivity after effortful emotion regulation in borderline personality disorder. Eur Arch Psychiatry Clin Neurosci. (2017) 267:551–65. doi: 10.1007/s00406-016-0760-z PMC5561271 28039553

[112] Chase HW Phillips ML. Elucidating neural network functional connectivity abnormalities in bipolar disorder: toward a harmonized methodological approach. Biol Psychiatry Cogn. Neurosci Neuroimaging. (2017) 2:1–22. doi: 10.1016/j.bpsc.2015.12.006 PMC4956344 27453953

[113] Strakowski SM Adler CM Almeida J Altshuler LL Blumberg HP Chang KD. The functional neuroanatomy of bipolar disorder: a consensus model. Bipolar Disord. (2012) 14:313–25. doi: 10.1111/j.1399-5618.2012.01022.x PMC3874804 22631617

[114] Bøen E Hjørnevik T Hummelen B Elvsåshagen T Moberget T Holtedahl JE. Patterns of altered regional brain glucose metabolism in borderline personality disorder and bipolar II disorder. Acta Psychiatr Scand. (2019) 139:256–68. doi: 10.1111/acps.2019.139.issue-3 30552759

[115] Rossi R Pievani M Lorenzi M Boccardi M Beneduce R Bignotti S. Structural brain features of borderline personality and bipolar disorders. Psychiatry Res - Neuroimaging. (2013) 213:83–91. doi: 10.1016/j.pscychresns.2012.07.002 23146251

[116] Aleknaviciute J Tulen JHM Kamperman AM de Rijke YB Kooiman CG Kushner SA. Borderline and cluster C personality disorders manifest distinct physiological responses to psychosocial stress. Psychoneuroendocrinology. (2016) 72:131–8. doi: 10.1016/j.psyneuen.2016.06.010 27413994

[117] Bourvis N Aouidad A Cabelguen C Cohen D Xavier J. How do stress exposure and stress regulation relate to borderline personality disorder? Front Psychol. (2017) 8:2054. doi: 10.3389/fpsyg.2017.02054 29250007 PMC5714931

[118] Duesenberg M Wolf OT Metz S Roepke S Fleischer J Elias V. Psychophysiological stress response and memory in borderline personality disorder. Eur J Psychotraumatol. (2019) 10:1568134. doi: 10.1080/20008198.2019.1568134 30788063 PMC6374976

[119] Wieck A Grassi-Oliveira R do Prado CH Rizzo LB de Oliveira AS Kommers- Molina J. Pro- inflammatory cytokines and soluble receptors in response to acute psychosocial stress: differential reactivity in bipolar disorder. Neurosci Lett. (2014) 580:17–21. doi: 10.1016/j.neulet.2014.07.040 25092610

[120] Lapomarda G Grecucci A Messina I Pappaianni E Dadomo H. Common and different gray and white matter alterations in bipolar and borderline personality disorder: A source-based morphometry study. Brain Res. (2021) 1762:147401. 33675742 10.1016/j.brainres.2021.147401

[121] Buhle JT Silvers JA Wager TD Lopez R Onyemekwu C Kober H. . 10.1093/cercor/bht154 PMC4193464 23765157

[122] Grecucci A Giorgetta C Bonini N Sanfey A. Reappraising social emotions: the role of inferior frontal gyrus, temporo-parietal junction and insula in interpersonal emotion regulation. Front Hum Neurosci. (2013) 7:523. 24027512 10.3389/fnhum.2013.00523 PMC3759791

[123] Grecucci A Giorgetta C van Wout M Bonini N Sanfey A. Reappraising the Ultimatum: an fMRI study of emotion regulation and decision-making. Cereb Cortex. (2013) 23:399–410. doi: 10.1093/cercor/bhs028 22368088

[124] Messina I Bianco S Sambin M Viviani R. Executive and semantic processes in reappraisal of negative stimuli: insights from a meta-analysis of neuroimaging studies. Front Psychol. (2015) 6:956. doi: 10.3389/fpsyg.2015.00956 26217277 PMC4499672

[125] Miskowiak KW Obel ZK Guglielmo R Bonnin C del M Bowie CR. Efficacy and safety of established and off-label ADHD drug therapies for cognitive impairment or attention-deficit hyperactivity disorder symptoms in bipolar disorder: A systematic review by the ISBD Targeting Cognition Task Force. Bipolar Disord. (2024) 26:216–39. doi: 10.1111/bdi.13414 38433530

[126] Schiweck C Arteaga-Henriquez G Aichholzer M Edwin Thanarajah S Vargas-Cáceres S Matura S. Comorbidity of ADHD and adult bipolar disorder: A systematic review and meta-analysis. Neurosci Biobehav Rev. (2021) 124:100–23. doi: 10.1016/j.neubiorev.2021.01.017 33515607

[127] Torres I Sole B Corrales M Jiménez E Rotger S Serra-Pla JF. Are patients with bipolar disorder and comorbid attention-deficit hyperactivity disorder more neurocognitively impaired? Bipolar Disord. (2017) 19:637–50. doi: 10.1111/bdi.12540 28941032

[128] Sankar A Ziersen SC Ozenne B Beaman EE Dam VH Fisher PM. Association of neurocognitive function with psychiatric hospitalization and socio-demographic conditions in individuals with bipolar and major depressive disorders. EClinicalMedicine. (2023) 58:101927. doi: 10.1016/j.eclinm.2023.101927 37007740 PMC10050788

[129] Lenzi F Cortese S Harris J Masi G. Pharmacotherapy of emotional dysregulation in adults with ADHD: A systematic review and meta-analysis. Neurosci Biobehav Rev. (2018) 84:359–67. doi: 10.1016/J.NEUBIOREV.2017.08.010 28837827

[130] Long Y Pan N Yu Y Zhang S Qin K Chen Y. Shared and distinct neurobiological bases of bipolar disorder and attention-deficit/hyperactivity disorder in children and adolescents: A comparative meta-analysis of structural abnormalities. J Am Acad Child Adolesc Psychiatry. (2024) 63:586–604. doi: 10.1016/j.jaac.2023.09.551 38072245

[131] Passarotti AM Sweeney JA Pavuluri MN. Differential engagement of cognitive and affective neural systems in pediatric bipolar disorder and attention deficit hyperactivity disorder. J Int Neuropsychol Soc. (2010) 16:106–17. doi: 10.1017/S1355617709991019 PMC3169194 19849880

[132] Hafeman D Bebko G Bertocci MA Fournier JC Chase HW Bonar L. Amygdala-prefrontal cortical functional connectivity during implicit emotion processing differentiates youth with bipolar spectrum from youth with externalizing disorders. J Affect Disord. (2017) 208:94–100. doi: 10.1016/j.jad.2016.09.064 27756046 PMC5154789

[133] Son YD Han DH Kim SM Min KJ Renshaw PF. A functional connectivity comparison between attention deficit hyperactivity disorder and bipolar disorder in medication-naïve adolescents with mood fluctuation and attention problems. Psychiatry Res Neuroimaging. (2017) 263:1–7. doi: 10.1016/j.pscychresns.2017.02.006 28264765

[134] Alda M. Lithium in the treatment of bipolar disorder: Pharmacology and mechanisms of action. Mol Psychiatry. (2015) 20:661–70. doi: 10.1038/mp.2015.4 PMC5125816 25687772

[135] Hidalgo-Mazzei D Mantingh T Pérez de Mendiola X Samalin L Undurraga J Strejilevich S. Clinicians’ preferences and attitudes towards the use of lithium in the maintenance treatment of bipolar disorders around the world: a survey from the ISBD Lithium task force. Int J Bipolar Disord. (2023) 11:20. doi: 10.1186/s40345-023-00301-y 37243681 PMC10220344

[136] Malhi GS Outhred T. Therapeutic mechanisms of lithium in bipolar disorder: recent advances and current understanding. CNS Drugs. (2016) 30:931–49. doi: 10.1007/s40263-016-0380-1 27638546

[137] Won E Kim YK. An oldie but goodie: lithium in the treatment of bipolar disorder through neuroprotective and neurotrophic mechanisms. Int J Mol Sci. (2017) 18:2679. doi: 10.3390/ijms18122679 29232923 PMC5751281

[138] Hartberg CB Jørgensen KN Haukvik UK Lange EH Nesvåg R Westlye LT. Lithium treatment and hippocampal subfields and amygdala volumes in bipolar disorder. Bipolar Disord. (2015) 17:496–506. doi: 10.1111/bdi.12295 25809287

[139] Selek S Nicoletti M Zunta-Soares GB Hatch JP Soares JC Kapczinski F. A longitudinal study of fronto-limbic brain structures in patients with bipolar I disorder during lithium treatment. J Affect Disord. (2013) 150:629–33. doi: 10.1016/j.jad.2013.04.020 23764385

[140] Kafantaris V Spritzer L Doshi V Saito E Szeszko PR. Changes in white matter microstructure predict lithium response in adolescents with bipolar disorder. Bipolar Disord. (2017) 19:587–94. doi: 10.1111/bdi.12544 28992395

[141] Lei D Li W Qin K Ai Y Tallman MJ Patino LR. Effects of short-term quetiapine and lithium therapy for acute manic or mixed episodes on the limbic system and emotion regulation circuitry in youth with bipolar disorder. Neuropsychopharmacology. (2023) 48(4):615–22. 10.1038/s41386-022-01463-6 PMC9938175 36229596

[142] Bergamelli E Del Fabro L Delvecchio G D’Agostino A Brambilla P. The impact of lithium on brain function in bipolar disorder: an updated review of functional magnetic resonance imaging studies. CNS Drugs. (2021) 35:1275–87. doi: 10.1007/s40263-021-00869-y PMC9537229 34773217

[143] Hamdani N Doukhan R Kurtlucan O Tamouza R Leboyer M. Immunity, inflammation, and bipolar disorder: diagnostic and therapeutic implications. Curr Psychiatry Rep. (2013) 15:387. doi: 10.1007/s11920-013-0387-y 23955004

[144] Wang T Lee S-Y Chen S-L Chung Y-L Li C-L Chang Y-H. The differential levels of inflammatory cytokines and BDNF among bipolar spectrum disorders. Int J Neuropsychopharmacol. (2016) 19. doi: 10.1093/ijnp/pyw012 PMC5006191 26865313

[145] Misiak B Bartoli F Carrà G Małecka M Samochowiec J Jarosz K. Chemokine alterations in bipolar disorder: a systematic review and meta-analysis. Brain Behav Immun. (2020) 88:870–7. doi: 10.1016/j.bbi.2020.04.013 32278851

[146] Kauer-Sant’Anna M Kapczinski F Andreazza AC Bond DJ Lam RW Young LT. Brain-derived neurotrophic factor and inflammatory markers in patients with early-vs. late-stage bipolar disorder. Int J Neuropsychopharmacol. (2009) 12:447–58. doi: 10.1017/S1461145708009310 18771602

[147] Goldstein BI Kemp DE Soczynska JK McIntyre RS. Inflammation and the phenomenology, pathophysiology, comorbidity, and treatment of bipolar disorder: a systematic review of the literature. J Clin Psychiatry. (2009) 70:9049. 10.4088/JCP.08r04505 19497250

[148] Moriarity DP Grehl MM Walsh RF Roos LG Slavich GM Alloy LB. A systematic review of associations between emotion regulation characteristics and inflammation. Neurosci Biobehav Rev. (2023) 150:105162. doi: 10.1016/j.neubiorev.2023.105162 37028579 PMC10425218

[149] Appleton AA Buka SL Loucks EB Gilman SE Kubzansky LD. Divergent associations of adaptive and maladaptive emotion regulation strategies with inflammation. Health Psychol. (2013) 32:748. doi: 10.1037/a0030068 23815767 PMC3793468

[150] Brown RL Shahane AD Chen MA Fagundes CP. Cognitive reappraisal and nasal cytokine production following experimental rhinovirus infection. Brain Behavior Immunity-Health. (2020) 1:100012. doi: 10.1016/j.bbih.2019.100012 32140685 PMC7057831

[151] Ellis EM Prather AA Grenen EG Ferrer RA. Direct and indirect associations of cognitive reappraisal and suppression with disease biomarkers. Psychol Health. (2019) 34:336–54. doi: 10.1080/08870446.2018.1529313 PMC6492247 30614281

[152] Lopez RB Brown RL Wu ELL Murdock KW Denny BT Heijnen C. Emotion regulation and immune functioning during grief: testing the role of expressive suppression and cognitive reappraisal in inflammation among recently bereaved spouses. Psychosom. Med. (2020) 82:2–9. 31634318 10.1097/PSY.0000000000000755

[153] Ospina LH Beck-Felts K Ifrah C Lister A Messer S Russo SJ. Inflammation and emotion regulation: Findings from the MIDUS II study. Brain Behavior Immunity-Health. (2022) 26:100536. doi: 10.1016/j.bbih.2022.100536 36247835 PMC9563642

[154] Renna ME Hoyt MA Ottaviani C Mennin DS. An experimental examination of worry and relaxation on cardiovascular, endocrine, and inflammatory processes. Psychoneuroendocrinology. (2020) 122:104870. doi: 10.1016/j.psyneuen.2020.104870 33010599 PMC7849652

[155] Renna ME. A review and novel theoretical model of how negative emotions influence inflammation: The critical role of emotion regulation. Brain Behav Immun Health. (2021) 18:100397. doi: 10.1016/j.bbih.2021.100397 34927103 PMC8649080

[156] Zoccola PM Figueroa WS Rabideau EM Woody A Benencia F. Differential effects of poststressor rumination and distraction on cortisol and C-reactive protein. Health Psychol. (2014) 33:1606–9. doi: 10.1037/hea0000019 24467256

[157] Petruso F Giff AE Milano BA De Rossi MM Saccaro LF. Inflammation and emotion regulation: a narrative review of evidence and mechanisms in emotion dysregulation disorders. Neuronal Signaling. (2023) 7:NS20220077. doi: 10.1042/NS20220077 38026703 PMC10653990

[158] Benedetti F Aggio V Pratesi ML Greco G Furlan R. Neuroinflammation in bipolar depression. FrontPsychiatry. (2020) 11:71. doi: 10.3389/fpsyt.2020.00071 PMC7054443 32174850

[159] Grande I Berk M Birmaher B Vieta E. Bipolar disorder. Lancet. (2016) 387:1561–72. doi: 10.1016/S0140-6736(15)00241-X 26388529

[160] Phillips M. The neural basis of mood dysregulation in bipolar disorder. Cognit Neuropsychiatry. (2006) 11:233–49. doi: 10.1080/13546800444000290 17354070

[161] Piguet C Karahanoğlu FI Saccaro LF Van De Ville D Vuilleumier P. Mood disorders disrupt the functional dynamics, not spatial organization of brain resting state networks. NeuroImage Clin. (2021) 32:102833. doi: 10.1016/j.nicl.2021.102833 34619652 PMC8498469

[162] Haarman BCM Burger H Doorduin J Renken RJ Sibeijn-Kuiper A Marsman J-BC. Volume, metabolites and neuroinflammation of the hippocampus in bipolar disorder – a combined magnetic resonance imaging and Positron Emission Tomography Study. Brain Behavior Immun. (2016) 56:21–33. doi: 10.1016/j.bbi.2015.09.004 26348581

[163] Tu P Li CT Lin WC Chen MH Su TP Bai YM. Structural and functional correlates of serum soluble IL-6 receptor level in patients with bipolar disorder. J Affect. Disord. (2017) 219:172–7. doi: 10.1016/j.jad.2017.04.036 28558364

[164] Dargél AA Godin O Etain B Hirakata V Azorin JM M’bailara K. Emotional reactivity, functioning, and C-reactive protein alterations in remitted bipolar patients: Clinical relevance of a dimensional approach. Aust New Z J Psychiatry. (2017) 51:788–98. doi: 10.1177/0004867417691850 28374603

[165] Dargél AA Roussel F Volant S Etain B Grant R Azorin JM. Emotional hyper-reactivity and cardiometabolic risk in remitted bipolar patients: a machine learning approach. Acta Psychiatrica Scandinavica. (2018) 138:348–59. doi: 10.1111/acps.12901 29766490

[166] Radaelli D Papa GS Vai B Poletti S Smeraldi E Colombo C. Fronto-limbic disconnection in bipolar disorder. Eur Psychiatry. (2015) 30:82–8. doi: 10.1016/j.eurpsy.2014.04.001 24853295

[167] Benedetti F Poletti S Hoogenboezem TA Mazza E Ambrée O de Wit H. Inflammatory cytokines influence measures of white matter integrity in bipolar disorder. J Affect Disord. (2016) 202:1–9. doi: 10.1016/j.jad.2016.05.047 27253210

[168] Poletti S de Wit H Mazza E Wijkhuijs AJ Locatelli C Aggio V. Th17 cells correlate positively to the structural and functional integrity of the brain in bipolar depression and healthy controls. Brain Behav Immun. (2017) 61:317–25. doi: 10.1016/j.bbi.2016.12.020 28025071

[169] Snijders G Brouwer R Kemner S Bootsman F Drexhage HA Hillegers MHJ. Genetic and environmental influences on circulating NK and T cells and their relation to bipolar disorder. Int J Bipolar Disord. (2019) 7:4. doi: 10.1186/s40345-018-0139-3 30739250 PMC6368934

[170] Furlan R Melloni E Finardi A Vai B Di Toro S Aggio V. Natural killer cells protect white matter integrity in bipolar disorder. Brain Behavior Immun. (2019) 81:410–21. doi: 10.1016/j.bbi.2019.06.037 31254622

[171] Brietzke E Mansur RB Soczynska JK Kapczinski F Bressan RA McIntyre RS. Towards a multifactorial approach for prediction of bipolar disorder in at risk populations. J Affect. Disord. (2012) 140:82–91. doi: 10.1016/j.jad.2012.02.016 22406334

[172] Altinoz MA Ozpinar A. Acetylsalicylic acid and its metabolite gentisic acid may act as adjunctive agents in the treatment of psychiatric disorders. Behav Pharmacol. (2019) 30:626–40. doi: 10.1097/FBP.0000000000000517 31703028

[173] Husain MI Strawbridge R Stokes PR Young AH. Anti-inflammatory treatments for mood disorders: systematic review and meta-analysis. J Psychopharmacol. (2017) 31:1137–48. doi: 10.1177/0269881117725711 28858537

[174] Rosenblat J McIntyre R. Bipolar disorder and immune dysfunction: epidemiological findings, proposed pathophysiology and clinical implications. Brain Sci. (2017) 7:144. doi: 10.3390/brainsci7110144 29084144 PMC5704151

[175] Rosenblat JD Kakar R Berk M Kessing LV Vinberg M Baune BT. Anti-inflammatory agents in the treatment of bipolar depression: a systematic review and meta-analysis. Bipolar Disord. (2016) 18:89–101. doi: 10.1111/bdi.2016.18.issue-2 26990051

[176] Knijff EM Nadine BM Kupka RW De Wit HJ Ruwhof C Akkerhuis GW. An imbalance in the production of IL-1β and IL-6 by monocytes of bipolar patients: restoration by lithium treatment. Bipolar Disord. (2007) 9:743–53. doi: 10.1111/j.1399-5618.2007.00444.x 17988365

[177] Padmos R Hillegers MHJ Knijff EM Vonk R Bouvy A Staal FJT. A discriminating messenger RNA signature for bipolar disorder formed by an aberrant expression of inflammatory genes in monocytes. Arch Gen Psychiatry. (2008) 65:395–407. doi: 10.1001/archpsyc.65.4.395 18391128

[178] Dargél AA Volant S Brietzke E Etain B Olié E Azorin JM. Allostatic load, emotional hyper-reactivity, and functioning in individuals with bipolar disorder. Bipolar Disord. (2020) 22:711–21. doi: 10.1111/bdi.12927 32415900

[179] Carruthers SP Rossell SL Murray G Karantonis J Furlong LS Van Rheenen TE. Mindfulness, mood symptom tendencies and quality of life in bipolar disorder: An examination of the mediating influence of emotion regulation difficulties. J Affect. Disord. (2022) 298:166–72. doi: 10.1016/j.jad.2021.10.107 34715199

[180] Murri MB Prestia D Mondelli V Pariante C Patti S Olivieri B. The HPA axis in bipolar disorder: systematic review and meta-analysis. Psychoneuroendocrinology. (2016) 63:327–42. doi: 10.1016/j.psyneuen.2015.10.014 26547798

[181] Mbiydzenyuy NE Qulu LA. Stress, hypothalamic-pituitary-adrenal axis, hypothalamic-pituitary-gonadal axis, and aggression. Metab Brain Dis. (2024) 39:1–24. doi: 10.1007/s11011-024-01393-w 39083184 PMC11535056

[182] Girshkin L Matheson SL Shepherd AM Green MJ. Morning cortisol levels in schizophrenia and bipolar disorder: a meta-analysis. Psychoneuroendocrinology. (2014) 49:187–206. doi: 10.1016/j.psyneuen.2014.07.013 25108162

[183] Juruena MF Cleare AJ Young AH. Neuroendocrine stress system in bipolar disorder. Bipolar Disorder: From Neuroscience to Treatment. (2021) 49–171. 10.1007/7854_2020_184 33580441

[184] Hoyt MA Marin-Chollom AM Bower JE Thomas KS Irwin MR Stan- ton AL. Approach and avoidance coping: diurnal cortisol rhythm in prostate cancer survivors. Psychoneuroendocrinology. (2014) 49:182–6. doi: 10.1016/j.psyneuen.2014.07.007 PMC4165795 25108161

[185] Denson TF Creswell JD Terides MD Blundell K. Cognitive reappraisal in- creases neuroendocrine reactivity to acute social stress and physical pain. Psychoneu- roendocrinology. (2014) 49:69–78. doi: 10.1016/j.psyneuen.2014.07.003 25063879

[186] Gilbert K Mineka S Zinbarg RE Craske MG Adam EK. Emotion regulation regulates more than emotion: associations of momentary emotion regulation with diurnal cortisol in current and past depression and anxiety. Clin Psychol Sci. (2017) 5:37–51. doi: 10.1177/2167702616654437 28944106 PMC5606207

[187] Nemeroff CB. Paradise lost: the neurobiological and clinical consequences of child abuse and neglect. Neuron. (2016) 89:892–909. doi: 10.1016/j.neuron.2016.01.019 26938439

[188] Jaffee SR. Child maltreatment and risk for psychopathology in childhood and adulthood. Annu Rev Clin Psychol. (2017) 13:525–51. doi: 10.1146/annurev-clinpsy-032816-045005 28375720

[189] Pechtel P Pizzagalli DA. Effects of early life stress on cognitive and affective function: an integrated review of human literature. Psychopharmacol (Berl.). (2011) 214:55–70. doi: 10.1007/s00213-010-2009-2 PMC3050094 20865251

[190] Cotter J Kaess M Yung AR. Childhood trauma and functional disability in psychosis, bipolar disorder and borderline personality disorder: a review of the literature. Irish J psychol Med. (2015) 32:21–30. doi: 10.1017/ipm.2014.74 30185274

[191] Hett D Etain B Marwaha S. Childhood trauma in bipolar disorder: new targets for future interventions. BJPsych Open. (2022) 8:e130. doi: 10.1192/bjo.2022.529 35815760 PMC9301771

[192] Etain B Lajnef M Brichant-Petitjean C Geoffroy PA Henry C Gard S. Childhood trauma and mixed episodes are associated with poor response to lithium in bipolar disorders. Acta Psychiatrica Scandinavica. (2017) 135:319–27. doi: 10.1111/acps.2017.135.issue-4 27987204

[193] Etain B Lajnef M Henry C Aubin V Azorin JM Bellivier F. Childhood trauma, dimensions of psychopathology and the clinical expression of bipolar disorders: a pathway analysis. J Psychiatr Res. (2017) 95:37–45. doi: 10.1016/j.jpsychires.2017.07.013 28777981

[194] Aas M Bellivier F Bettella F Henry C Gard S Kahn JP. Childhood maltreatment and polygenic risk in bipolar disorders. Bipolar Disord. (2020) 22:174–81. doi: 10.1111/bdi.12851 31628696

[195] Palmier-Claus J Golby R Stokes LJ Saville CW Velemis K Varese F. The relationship between childhood adversity and affective instability across psychiatric disorders: A meta-analysis. Acta Psychiatrica Scandinavica. (2024). doi: 10.1111/acps.13745 PMC11608813 39128865

[196] Aas M Aminoff SR Lagerberg TV Etain B Agartz I Andreassen OA. Affective lability in patients with bipolar disorders is associ- ated with high levels of childhood trauma. Psychiatry Res. (2014) 218:252–5. doi: 10.1016/j.psychres.2014.03.046 24803185

[197] Etain B Henry C Bellivier F Mathieu F Leboyer M. Beyond genetics: childhood affective trauma in bipolar disorder. Bipolar Disord. (2008) 10:867–76. doi: 10.1111/j.1399-5618.2008.00635.x 19594502

[198] Marwaha S Gordon-Smith K Broome M Briley PM Perry A Forty L. Affective instability, childhood trauma and major affective disorders. J Affect. Disord. (2016) 190:764–71. doi: 10.1016/j.jad.2015.11.024 26615365

[199] Numakawa T Yokomaku D Richards M Hori H Adachi N Kunugi H. Functional interactions between steroid hormones and neurotrophin BDNF. World J Biol Chem. (2010) 1:133. doi: 10.4331/wjbc.v1.i5.133 21540998 PMC3083963

[200] Alexander N Osinsky R Schmitz A Mueller E Kuepper Y Hennig J. The BDNF Val66Met polymorphism affects HPA-axis reactivity to acute stress. Psychoneuroendocrinology. (2010) 35:949–53. doi: 10.1016/j.psyneuen.2009.12.008 20079575

[201] Kowianski P Lietzau G Czuba E Waskow M Steliga A Morys J. BDNF: a key factor with multipotent impact on brain signaling and synaptic plasticity. Cell Mol Neurobiol. (2018) 38:579–93. doi: 10.1007/s10571-017-0510-4 PMC5835061 28623429

[202] Balaratnasingam S Janca A. Brain derived neurotrophic factor: a novel neurotrophin involved in psychiatric and neurological disorders. Pharmacol Ther. (2012) 134:116–24. doi: 10.1016/j.pharmthera.2012.01.006 22281237

[203] Grande I Fries GR Kunz M Kapczinski F. The role of BDNF as a mediator of neuroplasticity in bipolar disorder. Psychiatry Investig. (2010) 7:243–50. doi: 10.4306/pi.2010.7.4.243 PMC3022310 21253407

[204] Vega-Núñez A Gómez-Sánchez-Lafuente C Mayoral-Cleries F Bordallo A Rodríguez de Fonseca F Suárez J. Clinical value of inflammatory and neurotrophic biomarkers in bipolar disorder: a systematic review and meta-analysis. Biomedicines. (2022) 10:1368. doi: 10.3390/biomedicines10061368 35740389 PMC9220136

[205] Duffy A Goodday SM Keown-Stoneman C Scotti M Maitra M Nagy C. Epigenetic markers in inflammation-related genes associated with mood disorder: a cross-sectional and longitudinal study in high-risk offspring of bipolar parents. Int J Bipolar. Disord. (2019) 7:1–8. doi: 10.1186/s40345-019-0152-1 31385059 PMC6682840

[206] Cunha AB Frey BN Andreazza AC Goi JD Rosa AR Goncalves CA. Serum brain-derived neurotrophic factor is decreased in bipolar disorder during depressive and manic episodes. Neurosci Lett. (2006) 398:215–9. doi: 10.1016/j.neulet.2005.12.085 16480819

[207] de Oliveira GS Ceresér KM Fernandes BS Kauer-Sant’Anna M Fries GR Stertz L. Decreased brain-derived neurotrophic factor in medicated and drug-free bipolar patients. J Psychiatr Res. (2009) 43:1171–4. doi: 10.1016/j.jpsychires.2009.04.002 19473667

[208] MaChado-Vieira R Dietrich MO Leke R Cereser VH Zanatto V Kapczinski F. Decreased plasma brain derived neurotrophic factor levels in unmedicated bipolar patients during manic episode. Biol Psychiatry. (2007) 61:142–4. doi: 10.1016/j.biopsych.2006.03.070 16893527

[209] Fernandes BS Gama CS Kauer-Sant’Anna M Lobato MI Belmonte-de-Abreu P Kapczinski F. Serum brain-derived neurotrophic factor in bipolar and unipolar depression: a potential adjunctive tool for differential diagnosis. J Psychiatr Res. (2009) 43:1200–4. doi: 10.1016/j.jpsychires.2009.04.010 19501841

[210] Tramontina JF Andreazza AC Kauer-Sant’anna M Stertz L Goi J Chiarani F. Brain-derived neurotrophic factor serum levels before and after treatment for acute mania. Neurosci Lett. (2009) 452:111–3. doi: 10.1016/j.neulet.2009.01.028 19383424

[211] Perez-Rodriguez MM New AS Goldstein KE Rosell D Yuan Q Zhou Z. Brain-derived neurotrophic factor Val66Met genotype modulates amygdala habituation. Psychiatry Research: Neuroimaging. (2017) 263:85–92. doi: 10.1016/j.pscychresns.2017.03.008 28371657 PMC5856456

[212] Miu AC Cărnuţă M Vulturar R Szekely-Copîndean RD Bîlc MI Chiş A. BDNF Val66Met polymorphism moderates the link between child maltreatment and reappraisal ability. Genes Brain Behav. (2017) 16:419–26. doi: 10.1111/gbb.2017.16.issue-4 28009101

[213] Takaesu Y. Circadian rhythm in bipolar disorder: a review of the literature. Psychiatry Clin Neurosci. (2018) 72:673–82. doi: 10.1111/pcn.2018.72.issue-9 29869403

[214] Kishi T Kitajima T Ikeda M Yamanouchi Y Kinoshita Y Kawashima K. Association study of clock gene (CLOCK) and schizophrenia and mood disorders in the Japanese population. Eur Arch Psychiatry Clin Neurosci. (2009) 259:293–7. doi: 10.1007/s00406-009-0869-4 19224106

[215] Lee KY Song JY Kim SH Yoon HK Choi YK Jung HY. Association between CLOCK 3111T/C and preferred circadian phase in Korean patients with bipolar disorder. Prog Neuropsychopharmacol Biol Psychiatry. (2010) 34:1196–201. doi: 10.1016/j.pnpbp.2010.06.010 20600471

[216] Mansour HA Wood J Logue T Chowdari KV Dayal M Kupfer DJ. Association study of eight circadian genes with bipolar I disorder, schizoaffective disorder and schizophrenia. Genes Brain Behav. (2006) 5:150–7. doi: 10.1111/j.1601-183X.2005.00147.x 16507006

[217] Nievergelt CM Kripke DF Barrett TB Burg E Remick RA Sadovnick AD. Suggestive evidence for association of the circadian genes PERIOD3 and ARNTL with bipolar disorder. Am J Med Genet B Neuropsychiatr Genet. (2006) 141B:234–41. doi: 10.1002/ajmg.b.30252 PMC2651679 16528748

[218] Shi J Wittke-Thompson JK Badner JA Hattori E Potash JB Willour VL. Clock genes may influence bipolar disorder susceptibility and dysfunctional circadian rhythm. Am J Med Genet B Neuropsychiatr Genet. (2008) 147B:1047–55. doi: 10.1002/ajmg.b.30714 PMC2574897 18228528

[219] Drago A Monti B De Ronchi D Serretti A. CRY1 variations impacts on the depressive relapse rate in a sample of bipolar patients. Psychiatry Invest. (2015) 12:118. doi: 10.4306/pi.2015.12.1.118 PMC4310909 25670954

[220] Titone MK Goel N Ng TH MacMullen LE Alloy LB. Impulsivity and sleep and circadian rhythm disturbance predict next-day mood symptoms in a sample at high risk for or with recent-onset bipolar spectrum disorder: An ecological momentary assessment study. J Affect Disord. (2022) 298:17–25. doi: 10.1016/j.jad.2021.08.155 34728283 PMC8643329

[221] Alloy LB Walsh RF Smith LT Maddox MA Olino TM Zee PC. Circadian, Reward, and Emotion Systems in Teens prospective longitudinal study: protocol overview of an integrative reward-circadian rhythm model of first onset of bipolar spectrum disorder in adolescence. BMC Psychiatry. (2023) 23:602. doi: 10.1186/s12888-023-05094-z 37592214 PMC10436678

[222] Reppert SM Wever DR. Molecular analysis of mammalian circadian rhythms. Ann Rev Physiol. (2001) 63:647–76. doi: 10.1146/annurev.physiol.63.1.647 11181971

[223] Roenneberg T Merrow M. Entrainment of the human circadian clock. Cold Spring Harb Symp Quant Biol. (2007) 72:293–9. 10.1101/sqb.2007.72.043 18419286

[224] Jagannath A Taylor L Wakaf Z Vasudevan SR Foster RG. The genetics of circadian rhythms, sleep and health. Hum Mol Genet. (2017) 26:R128–38. doi: 10.1093/hmg/ddx240 PMC5886477 28977444

[225] Melo MC Garcia RF Linhares Neto VB Sa MB de Mesquita LM de Araujo CF. Sleep and circadian alterations in people at risk for bipolar disorder: a systematic review. J Psychiatr Res. (2016) 83:211–9. doi: 10.1016/j.jpsychires.2016.09.005 27661417

[226] Robillard R Naismith SL Rogers NL Scott EM Ip TKC Hermens DF. Sleep-wake cycle and melatonin rhythms in adolescents and young adults with mood disorders: comparison of unipolar and bipolar phenotypes. Eur Psychiat. (2013) 28:412–6. doi: 10.1016/j.eurpsy.2013.04.001 23769680

[227] Robillard R Hermens DF Naismith SL White D Rogers NL Ip TK. Ambulatory sleep-wake patterns and variability in young people with emerging mental disorders. J Psychiatry Neurosci. (2015) 40:28–37. doi: 10.1503/jpn.130247 25203899 PMC4275328

[228] Smagula SF Krafty RT Thayer JF Buysse DJ Hall MH. Rest-activity rhythm profiles associated with manic-hypomanic and depressive symptoms. J Psychiatr Res. (2018) 102:238–44. doi: 10.1016/j.jpsychires.2018.04.015 PMC6005763 29705489

[229] Steinan MK Scott J Lagerberg TV Melle I Andreassen OA Vaaler AE. Sleep problems in bipolar disorders: more than just insomnia. Acta Psychiatrica Scandinavica. (2016) 133:368–77. doi: 10.1111/acps.12523 PMC5063196 26590799

[230] Glozier N O’Dea B McGorry PD Pantelis C Amminger GP Hermens DF. Delayed sleep onset in depressed young people. BMC Psychiatry. (2014) 14:1–9. doi: 10.1186/1471-244X-14-33 PMC3938136 24506941

[231] Zhang J Paksarian D Lamers F Hickie IB He J Merikangas KR. Sleep patterns and mental health correlates in US adolescents. J Pediatr. (2017) 182:137–43. 10.1016/j.jpeds.2016.11.007 27939122

[232] Logan RW McClung CA. Rhythms of life: Circadian disruption and brain disorders across the lifespan. Nat Rev Neurosci. (2019) 20:49–65. 30459365 10.1038/s41583-018-0088-y PMC6338075

[233] Faria AD de Azevedo Cardoso T Mondin TC de Mattos Souza LD da Silva Magalhaes PV Zeni CP. Biological rhythms in bipolar and depressive disorders: a community study with drug-naïve young adults. J Affect Disord. (2015) 186:145–8. doi: 10.1016/j.jad.2015.07.004 26241662

[234] Mondin TC de Azevedo Cardoso T de Mattos Souza LD Jansen K da Silva Magalhães PV Kapczinski F. Mood disorders and biological rhythms in young adults: a large population-based study. J Psychiatr Res. (2017) 84:98–104. doi: 10.1016/j.jpsychires.2016.09.030 27716514

[235] Altena E Micoulaud-Franchi J-A Geoffroy P-A Sanz-Arigita E Bioulac S Philip P. The bidirectional relation between emotional reactivity and sleep: From disruption to recovery. Behav Neurosci. (2016) 130:336–50. doi: 10.1037/bne0000128 26866361

[236] Baglioni C Spiegelhalder K Lombardo C Riemann D. Sleep and emotions: a focus on insomnia. Sleep Med Rev. (2010) 14:227–38. doi: 10.1016/j.smrv.2009.10.007 20137989

[237] Kanady JC Soehnera AM Harvey AGA. Retrospective examination of sleep disturbance across the course of bipolar disorder. J Sleep Disord Ther. (2015) 30:4. doi: 10.4172/2167-0277.1000193 PMC4662555 26618073

[238] Krause AJ Simon EB Mander BA Greer SM Saletin JM Goldstein-Piekarski An. The sleep-deprived human brain. Nat Rev Neurosci. (2017) 18:404–18. doi: 10.1038/nrn.2017.55 PMC6143346 28515433

[239] Riemann D Nissen C Palagini L Otte A Perlis ML Spiegelhalder K. The neurobiology, investigation, and treatment of chronic insomnia. Lancet Neurol. (2015) 14:547–58. doi: 10.1016/S1474-4422(15)00021-6 25895933

[240] Yoo SS Gujar N Hu P Jolesz FA Walker MP. The human emotional brain without sleep—a prefrontal amygdala disconnect. Curr Biol. (2007) 17:877–8. doi: 10.1016/j.cub.2007.08.007 17956744

[241] Rossa KR Smith SS Allan AC Sullivan KA. The effects of sleep restriction on executive inhibitory control and affect in young adults. J Adolesc Health. (2014) 55:287–92. doi: 10.1016/j.jadohealth.2013.12.034 24602612

[242] Van Someren EJ Cirelli C Dijk DJ Van Cauter E Schwartz S Chee MW. Disrupted sleep: from molecules to cognition. J Neurosci. (2015) 35:13889–95. doi: 10.1523/JNEUROSCI.2592-15.2015 PMC4604227 26468189

[243] Geoffroy PA Scott J Boudebesse C Lajnef M Henry C Leboyer M. Sleep in patients with remitted bipolar dis- orders: a meta-analysis of actigraphy studies. Acta Psychiatr Scand. (2015) 131:89–99. doi: 10.1111/acps.2014.131.issue-2 25430914

[244] Ritter PS Marx C Bauer M Leopold K Pfennig A. The role of disturbed sleep in the early recognition of bipolar disorder: a systematic review. Bipolar Disord. (2011) 13:227–37. doi: 10.1111/j.1399-5618.2011.00917.x 21676126

[245] Ritter PS Marx C Lewtschenko N Pfeiffer S Leopold K Bauer M. The characteristics of sleep in patients with manifest bipolar disorder, subjects at high risk of developing the disease and healthy controls. J Neural Transm. (2012) 119:1173–84. doi: 10.1007/s00702-012-0883-y 22903311

[246] Ritter PS Höfler M Wittchen HU Lieb R Bauer M Pfenning A. Disturbed sleep as risk factor for the subsequent onset of bipolar disorder—data from a 10-year prospective-longitudinal study among adolescents and young adults. J Psychiatr Res. (2015) 68:76–82. doi: 10.1016/j.jpsychires.2015.06.005 26228404

[247] Boudebesse C Henry C. Emotional hyper-reactivity and sleep disturbances in remitted patients with bipolar disorders. Encéphale. (2012) 38:173–8. doi: 10.1016/S0013-7006(12)70096-9 23395233

[248] Etain B Godin O Boudebesse C Aubin V Azorin JM Bellivier F. Sleep quality and emotional reactivity cluster in bipolar disorders and impact on functioning. European Psychiatry. (2017) 45:190–7. 10.1016/j.eurpsy.2017.06.013 28957786

[249] Horne CM Norbury R. Altered resting-state connectivity within default mode network associated with late chronotype. J Psychiatr Res. (2018) 102:223–9. doi: 10.1016/j.jpsychires.2018.04.013 29702432

[250] Kitamura S Hida A Watanabe M Enomoto M Aritake-Okada S Moriguchi Y. Evening prefer- ence is related to the incidence of depressive states inde- pendent of sleep-wake conditions. Chronobiol. Int. (2010) 27:1797–812. 10.3109/07420528.2010.516705 20969524

[251] Takeuchi H Taki Y Sekiguchi A Nouchi R Kotozaki Y Nakagawa S. Regional gray matter density is associated with morningness– eveningness: evidence from voxel-based morphometry. Neuroimage. (2015) 117:294–304. doi: 10.1016/j.neuroimage.2015.05.037 26003859

[252] Santos IM Bem-Haja P Silva A Rosa C Queiroz DF Alves MF. The interplay between chronotype and emotion regulation in the recognition of facial expressions of emotion. Behav Sci. (2022) 13:38. doi: 10.3390/bs13010038 36661610 PMC9855169

[253] Gershon A Kaufmann CN Depp CA Miller S Do D Zeitzer JM. Subjective versus objective evening chronotypes in bipolar disorder. J Affect. Disord. (2018) 225:342–9. doi: 10.1016/j.jad.2017.08.055 PMC5626649 28843917

[254] Kaufmann CN Gershon A Depp CA Miller S Zeitzer JM Ketter TA. Daytime midpoint as a digital biomarker for chronotype in bipolar disorder. J Affect Disord. (2018) 241:586–91. doi: 10.1016/j.jad.2018.08.032 PMC6436809 30172210

[255] Koskenvuo M Hublin C Partinen M Heikkilä K Kaprio J. Heritability of diurnal type: a nationwide study of 8753 adult twin pairs. J sleep Res. (2007) 16:156–62. doi: 10.1111/j.1365-2869.2007.00580.x 17542945

[256] Vink JM Vink JM Groot AS Kerkhof GA Boomsma DI. Genetic analysis of morningness and eveningness. Chronobiology Int. (2001) 18:809–22. doi: 10.1081/CBI-100107516 11763988

[257] Xu N Shinohara K Saunders KE Geddes JR Cipriani A. Effect of lithium on circadian rhythm in bipolar disorder: A systematic review and meta-analysis. Bipolar Disord. (2021) 23:445–53. doi: 10.1111/bdi.13070 33650218

[258] Zou H Zhou H Yan R Yao Z Lu Q. Chronotype, circadian rhythm, and psychiatric disorders: Recent evidence and potential mechanisms. Front Neurosci. (2022) 16:811771. doi: 10.3389/fnins.2022.811771 36033630 PMC9399511

[259] McCarthy MJ Nievergelt CM Kelsoe JR Welsh DK. A survey of genomic studies supports association of circadian clock genes with bipolar disorder spectrum illnesses and lithium response. PloS One. (2012) 7:e32091. doi: 10.1371/journal.pone.0032091 22384149 PMC3285204

[260] Pisanu C Congiu D Melis C Severino G Angius A Ardau R. Involvement of core clock genes in lithium response. World J Biol Psychiatry. (2017), 1–2. 28649929 10.1080/15622975.2017.1346281

[261] Yoshikawa T Honma S. Lithium lengthens circadian period of cultured brain slices in area specific manner. Behav Brain Res. (2016) 314:30–7. doi: 10.1016/j.bbr.2016.07.045 27478137

[262] Yin L Wang J Klein PS Lazar MA. Nuclear receptor Rev-erbα is a critical lithium-sensitive component of the circadian clock. Science. (2006) 311:1002–5. doi: 10.1126/science.1121613 16484495

[263] Chung S Lee EJ Yun S Choe HK Park SB Son HJ. Impact of circadian nuclear receptor REV-ERBα on midbrain dopamine production and mood regulation. Cell. (2014) 157:858–68. doi: 10.1016/j.cell.2014.03.039 24813609

[264] Kim J Jang S Choe HK Chung S Son GH Kim K. Implications of circadian rhythm in dopamine and mood regulation. Molecules Cells. (2017) 40:450–6. doi: 10.14348/molcells.2017.0065 PMC5547214 28780783

[265] Berking M Wupperman P. Emotion regulation and mental health: Recent findings, current challenges, and future directions. Curr Opin Psychiatry. (2012) 25:128–34. doi: 10.1097/YCO.0b013e3283503669 22262030

[266] Gruber J Johnson SL Oveis C Keltner D. Risk for mania and positive emotional responding: Too much of a good thing? Emotion. (2014) 8:23–33. 10.1037/1528-3542.8.1.23 PMC2847501 18266513

[267] Thompson ER Wright AG Gotlib IH. Assessment of emotion regulation: Theories, measures, and translational challenges. psychol Bull. (2020) 146:321–49.

[268] Van Rheenen TE Murray G Rossell SL. Emotion regulation in bipolar disorder: Profiling strengths and weaknesses. J Affect Disord. (2020) 273:124–34.

[269] Yatham LN Kennedy SH Parikh SV Schaffer A Bond DJ Frey BN. Canadian Network for Mood and Anxiety Treatments (CANMAT) and International Society for Bipolar Disorders (ISBD) 2018 guidelines for the management of patients with bipolar disorder. Bipolar Disord. (2018) 20:97–170. 29536616 10.1111/bdi.12609 PMC5947163

[270] Brietzke E Mansur RB Subramaniapillai M Balanzá-Martínez V Vinberg M González-Pinto A. Ketogenic diet as a metabolic therapy for mood disorders: evidence and developments. Neurosci Biobehav Rev. (2018) 94:11–6. doi: 10.1016/j.neubiorev.2018.07.020 30075165

[271] Needham N Campbell IH Grossi H Kamenska I Rigby BP Simpson SA. Pilot study of a ketogenic diet in bipolar disorder. BJPsych Open. (2023) 9:e176. doi: 10.1192/bjo.2023.568 37814952 PMC10594182

[272] Giménez-Palomo A Dodd S Anmella G Carvalho AF Scaini G Quevedo J. The role of mitochondria in mood disorders: from physiology to pathophysiology and to treatment. Front Psychiatry. (2021) 12:546801. doi: 10.3389/fpsyt.2021.546801 34295268 PMC8291901

[273] Sethi S Ford JM. The role of ketogenic metabolic therapy on the brain in serious. mental illness: a review. J Psychiatr Brain Sci. (2022) 7:e220009. 36483840 10.20900/jpbs.20220009 PMC9728807

[274] Koppel SJ Swerdlow RH. Neuroketotherapeutics: a modern review of a century-old therapy. Neurochemistry Int. (2018) 117:114–25. doi: 10.1016/j.neuint.2017.05.019 PMC5711637 28579059

[275] Sethi S Wakeham D Ketter T Hooshmand F Bjornstad J Richards B. Ketogenic diet intervention on metabolic and psychiatric health in bipolar and schizophrenia: A pilot trial. Psychiatry Res. (2024) 335:115866. doi: 10.1016/j.psychres.2024.115866 38547601

[276] Brenda JY Oz RS Sethi S. Ketogenic diet as a metabolic therapy for bipolar disorder: Clinical developments. J Affect Disord Rep. (2023) 11:100457.

[277] Ancoli-Israel S Martin JL Blackwell T Buenaver L Liu L Meltzer LJ. The SBSM guide to actigraphy monitoring: clinical and research applications. Behav Sleep Med. (2015) 13:S4–S38. doi: 10.1080/15402002.2015.1046356 26273913

[278] Zinkhan M Kantelhardt JW. Sleep assessment in large cohort studies with high-resolution accelerometers. Sleep Med Clin. (2016) 11:469–88. doi: 10.1016/j.jsmc.2016.08.006 28118871

[279] Krane-Gartiser K Steinan MK Langsrud K Vestvik V Sand T Fasmer OB. Mood and motor activity in euthymic bipolar disorder with sleep disturbance. J Affect. Disord. (2016) 202:23–31. 27253213 10.1016/j.jad.2016.05.012

[280] Urbanek JK Spira AP Di J Leroux A Crainiceanu C Zipunnikov V. Epidemiology of objectively measured bedtime and chronotype in US adolescents and adults: NHANES 2003-2006. Chronobiol Int. (2018) 35:416–34. 10.1080/07420528.2017.1411359 PMC5862775 29283283

[281] Matthews CE George SM Moore SC Bowles HR Blar A Park Y. Amount of time spent in sedentary behaviors and cause-specific mortality in US adults. Am J Clin Nutr. (2012) 95:437–45. doi: 10.3945/ajcn.111.019620 PMC3260070 22218159

[282] Doherty A Jackson D Hammerla N Plotz T Olivier P Granat MH. Large scale population assessment of physical activity using wrist worn accelerometers: The UK Biobank Study. PloS One. (2017) 12:e0169649. doi: 10.1371/journal.pone.0169649 28146576 PMC5287488

[283] Merikangas KR Swendsen J Hickie IB Cui L Shou H Merikangas AK. Real-time mobile monitoring of the dynamic associations among motor activity, energy, mood, and sleep in adults with bipolar disorder. JAMA Psychiatry. (2019) 76:190–8. doi: 10.1001/jamapsychiatry.2018.3546 PMC6439734 30540352

[284] Burton C McKinstry B Tătar AS Serrano-Blanco A Pagliari C Wolters M. Activity monitoring in patients with depression: a systematic review. J Affect Disord. (2013) 145:21–8. doi: 10.1016/j.jad.2012.07.001 22868056

[285] De Crescenzo F Economou A Sharpley AL Gormez A Quested DJ. Actigraphic features of bipolar disorder: a systematic review and meta-analysis. Sleep Med Rev. (2017) 33:58–69. doi: 10.1016/j.smrv.2016.05.003 28185811

[286] Lyall LM Wyse CA Graham N Ferguson A Lyall DM Cullen B. Association of disrupted circadian rhythmicity with mood disorders, subjective wellbeing, and cognitive function: a cross-sectional study of 91 105 participants from the UK Biobank. Lancet Psychiatry. (2018) 5:507–14. doi: 10.1016/s2215-0366(18)30139-1 29776774

[287] Bauer M Andreassen OA Geddes JR Kessing LV Lewitzka U Schulze TG. Areas of uncertainties and unmet needs in bipolar disorders: clinical and research perspectives. Lancet Psychiatry. (2018) 5:930–9. doi: 10.1016/S2215-0366(18)30253-0 30146246

[288] Vieta E Berk M Schulze TG Carvalho AF Suppes T Calabrese JR. Bipolar disorders. Nat Rev Dis Primers. (2018) 4:1–16. doi: 10.1038/nrdp.2018.8 29516993

[289] O’Leary E Dodd S Berk M. Biomarkers for bipolar disorder. In: Biomarkers in neuropsychiatry: A primer. Springer International Publishing, Cham (2023). p. 219–31.

[290] Hay AC Sheppes G Gross JJ Gruber J. Choosing how to feel: Emotion regulation choice in bipolar disorder. Emotion. (2015) 15:139–45. 10.1037/emo0000024 25313669

[291] Morris RW Sparks A Mitchell PB Weickert CS Green MJ. Lack of cortico-limbic coupling in bipolar disorder and schizophrenia during emotion regulation. Trans Psychiatry. (2012) 2:e90–0. 10.1038/tp.2012.16 PMC3309531 22832855

[292] Broch-Due I Kjærstad HL Kessing LV Miskowiak K. Subtle behavioural responses during negative emotion reactivity and down-regulation in bipolar disorder: A facial expression and eye-tracking study. Psychiatry Res. (2018) 266:152–9. 10.1016/j.psychres.2018.04.054 29864615

[293] Garcia-Blanco AC Perea M Salmeron L. Attention orienting and inhibitory control across the different mood states in bi- polar disorder: an emotional antisaccade task. Biol Psychol. (2013) 94:556–61. 10.1016/j.biopsycho.2013.10.005 24161800

[294] Soncin S Brien DC Coe BC Marin A Munoz DP. Contrasting emotion processing and executive functioning in attention-defi- cit/hyperactivity disorder and bipolar disorder. Behav Neurosci. (2016) 130:531–43. 10.1037/bne0000158 27537826

[295] Yep R Soncin S Brien DC Coe BC Marin A Munoz DP. Using an emotional saccade task to characterize executive functioning and emotion processing in attention-deficit hyperactivity disorder and bipolar disorder. Brain Cogn. (2018) 124:1–13. 29698907 10.1016/j.bandc.2018.04.002

[296] Berk M. Biomarkers in psychiatric disorders: status quo, impediments and facilitators. World Psychiatry. (2023) 22:174. 37159364 10.1002/wps.21071 PMC10168162

[297] Carvalho AF Köhler CA Fernandes BS Quevedo J Miskowiak KW Brunoni AR. Bias in emerging biomarkers for bipolar disorder. psychol Med. (2016) 46:2287–97. 10.1017/S0033291716000957 27193198

[298] Manchia M Vieta E Smeland OB Altimus C Bechdolf A Bellivier F. Translating big data to better treatment in bipolar disorder-a manifesto for coordinated action. Eur Neuropsychopharmacol. (2020) 36:121–36. 10.1016/j.euroneuro.2020.05.006 32536571

[299] Vallarino M Henry C Etain B Gehue LJ Macneil C Scott EM. An evidence map of psychosocial interventions for the earliest stages of bipolar disorder. Lancet Psychiatry. (2015) 2:548–63. 10.1016/S2215-0366(15)00156-X PMC4629930 26360451

[300] Vieta E Angst J. Bipolar disorder cohort studies: Crucial, but underfunded. Eur Neuropsychopharmacol. (2021) 47:3–5. doi: 10.1016/j.euroneuro.2021.03.008 33895615

[301] Mansur RB Lee Y McIntyre RS Brietzke E. What is bipolar disorder? A disease model of dysregulated energy expenditure. Neurosci Biobehav Rev. (2020) 113:529–45. 10.1016/j.neubiorev.2020.04.006 32305381

[302] Harrison PJ Colbourne L Harrison CH. The neuropathology of bipolar disorder: systematic review and meta-analysis. Mol Psychiatry. (2020) 25:1787–808. 10.1038/s41380-018-0213-3 PMC6292507 30127470

[303] Scangos KW State MW Miller AH Baker JT Williams LM. New and emerging approaches to treat psychiatric disorders. Nat Med. (2023) 29:317–33. 10.1038/s41591-022-02197-0 PMC11219030 36797480

